# Supplementary material for: Haploinsufficient phenotypes promote selection of PTEN and ARID1A-deficient clones in human colon
Source: EMBO Rep. 2025 Feb 7;26(5):1269–89. doi: 10.1038/s44319-025-00373-0 (PMC11893880; doi:10.1038/s44319-025-00373-0)
Supplement: Supplementary file 1 — Appendix [file 44319_2025_373_MOESM1_ESM.pdf]

Appendix for “Haploinsufficient phenotypes promote selection of PTEN and ARID1A-deficient clones in human colon”

Table of Contents

Appendix Figure S1 2
Appendix Figure S2 3
Appendix Figure S3 4
Appendix Figure S4 5
Appendix Table S1 6
Appendix Table S2 10
Appendix Table S3 11
Appendix Table S4 12

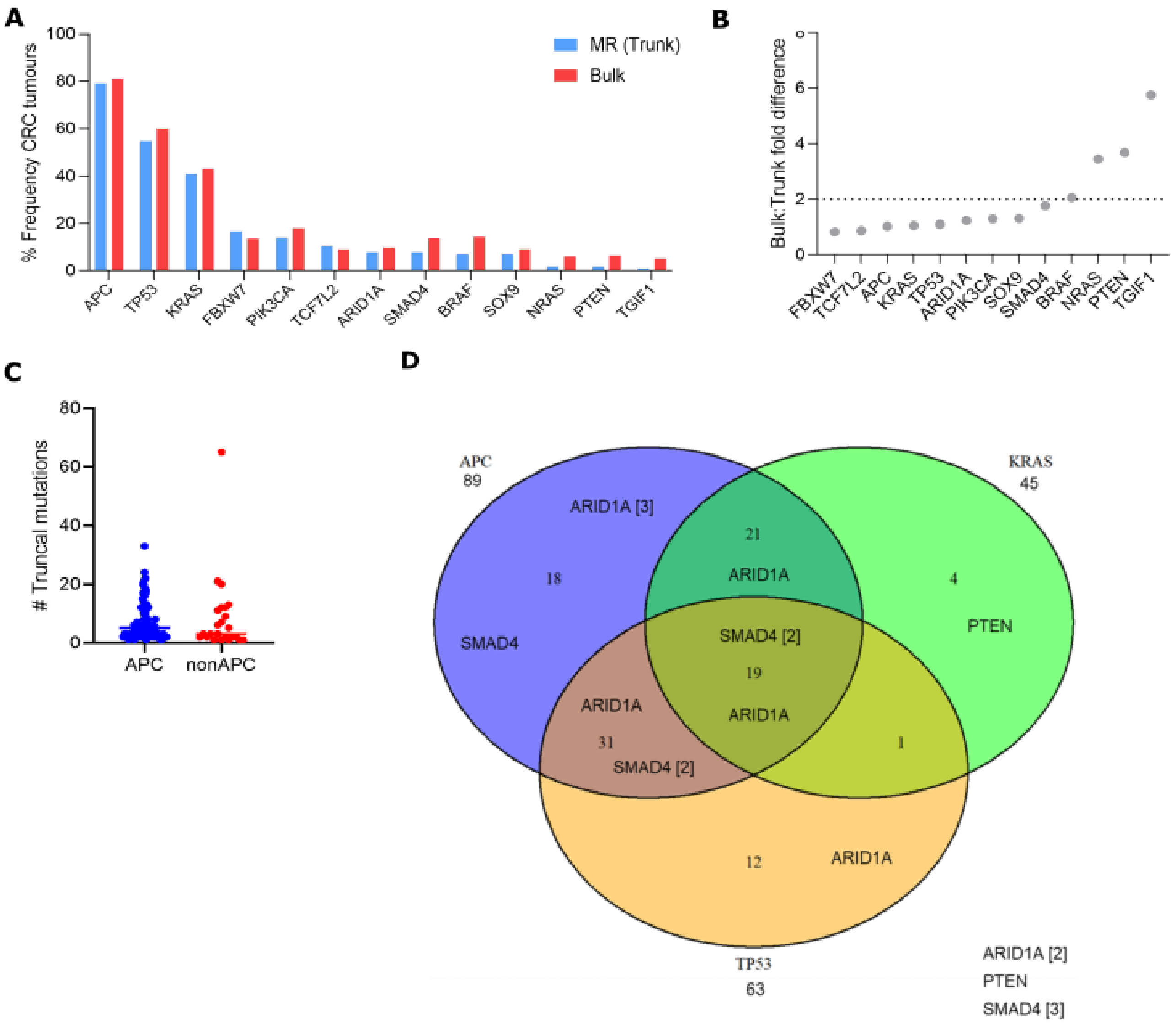

Appendix Figure S1: Metanalysis of truncal mutations identified using multi-regional sampling approaches. (A) Comparison of mutation frequency from the trunk of phylogenetic trees created from multi-regional studies or bulk sequencing (TCGA data). Includes genes with dN/dS >1 (q <0.001 Martincorena et al., 2017 CRC). (B) Fold difference in bulk versus trunk mutation frequency. 1:1 ratio would suggest early events and larger deviance from that would suggest higher frequency as a subclonal event. (C) Number of truncal mutations in APC or non-APC driven tumours. Considers mutations in all detected genes. (D) Venn diagram of relationship between APC, TP53 and KRAS driven tumours. PTEN, SMAD4 and ARID1A mutations annotated. Multiregional studies, N=115 tumours.

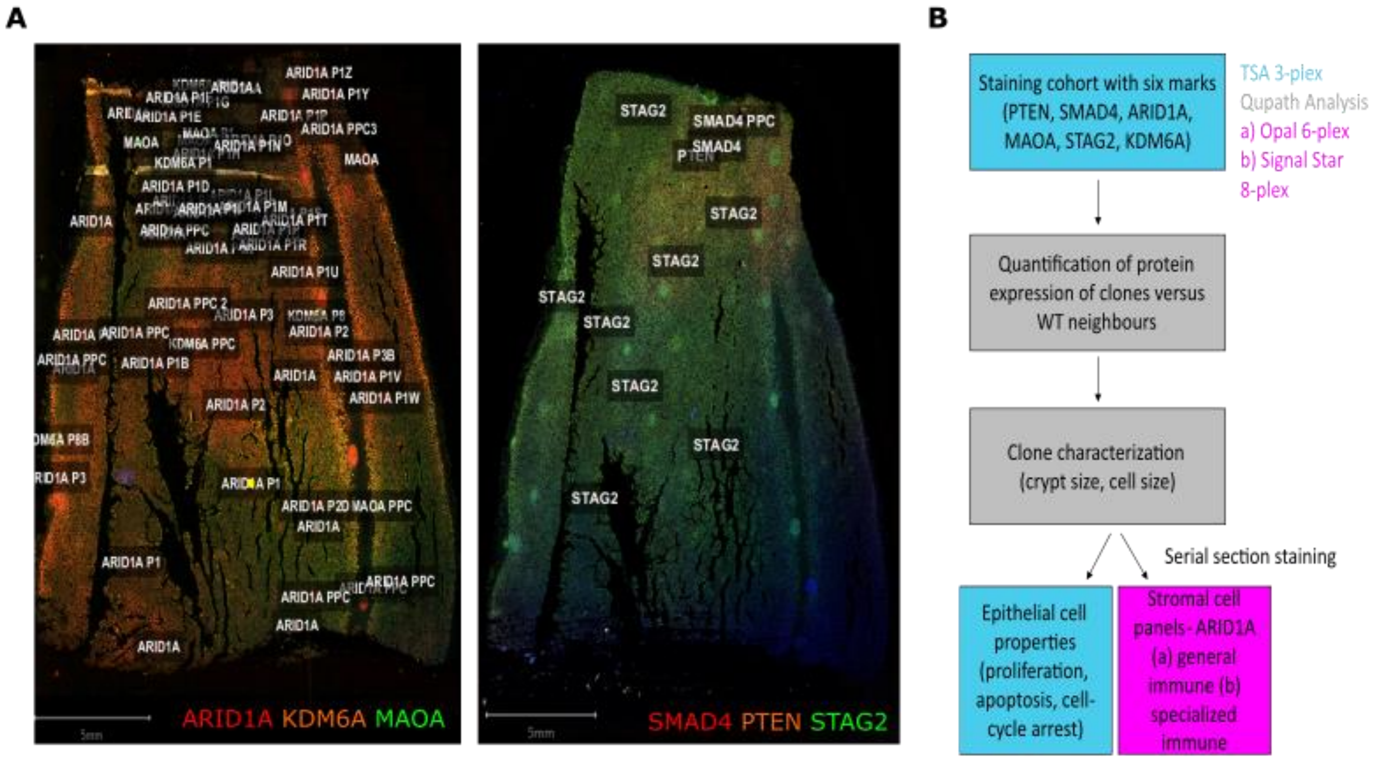

Appendix Figure S2: Use of sections for multiplexing immunofluorescence. (A) Whole slide imaging and annotation of clones. Stained as two 3-plex panels over two serial sections. (B) Layout of use of sections for selected patient cohort and application of multiplexing panels.

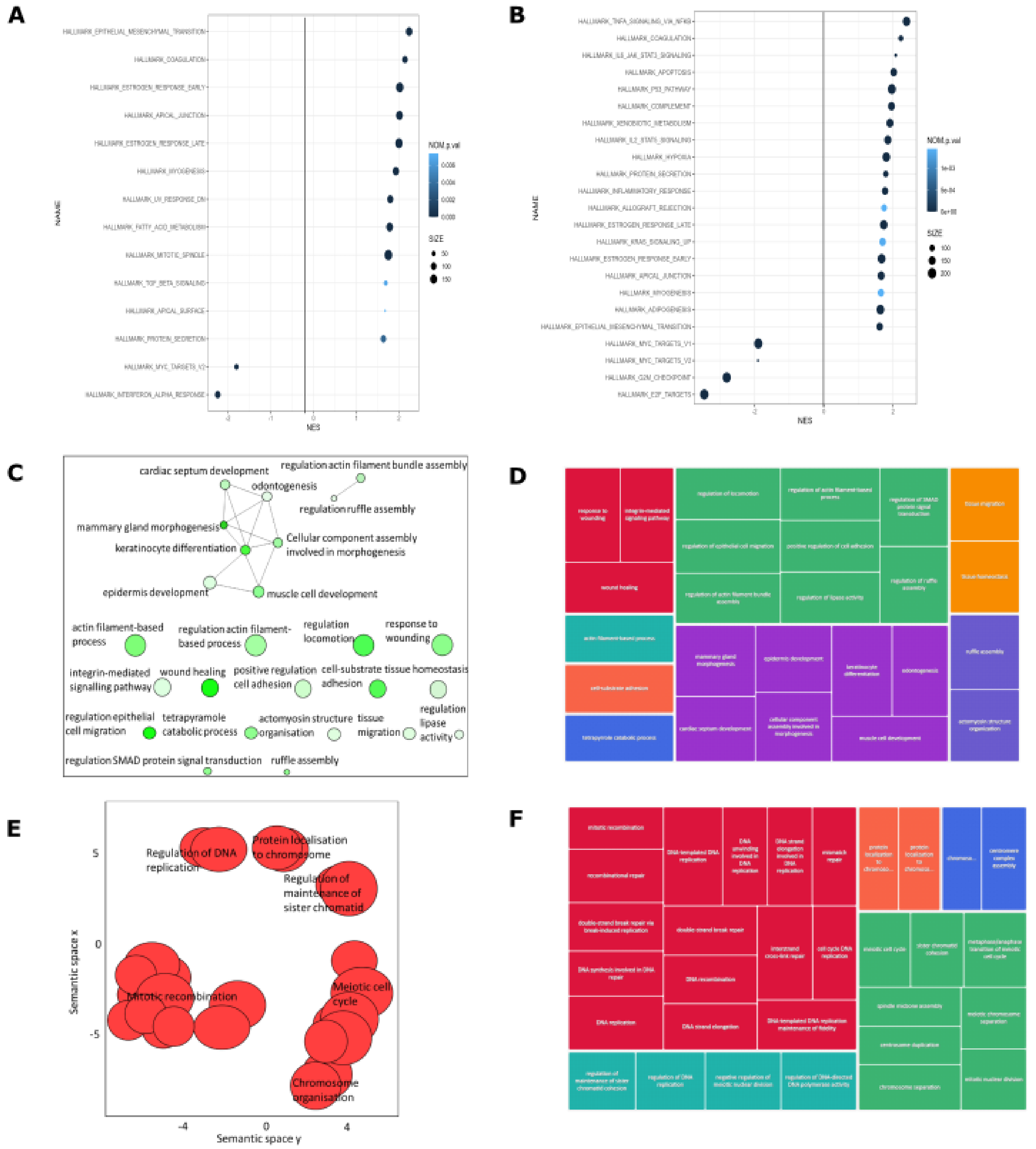

Appendix Figure S3: Gene set enrichment analysis (GSEA) for PTEN het and ARID1A het human colonic organoids vs WT. Considering gene sets FDR q-value < 1%. (A, C, D) PTEN het vs WT. (B, E, F) ARID1A het vs WT. (A-B) GSEA for hallmark pathways. (C) Interaction graph of positively enriched Gene Ontology Biological Processes (GOBP) generated in Revigo. Size of dot indicates logsizeof dataset and darker colour indicates higher enrichment score. (D, F) Tree map generated in Revigo. Cluster representatives (remaining terms after redudancy removal) are joined in high level groups. (E) Scatterplot showing cluster representatives in a two dimensional space derived by applying multidimensional scaling to a matrix of the GOBP term semantic similarities. Negatively enriched GOBP gene sets.

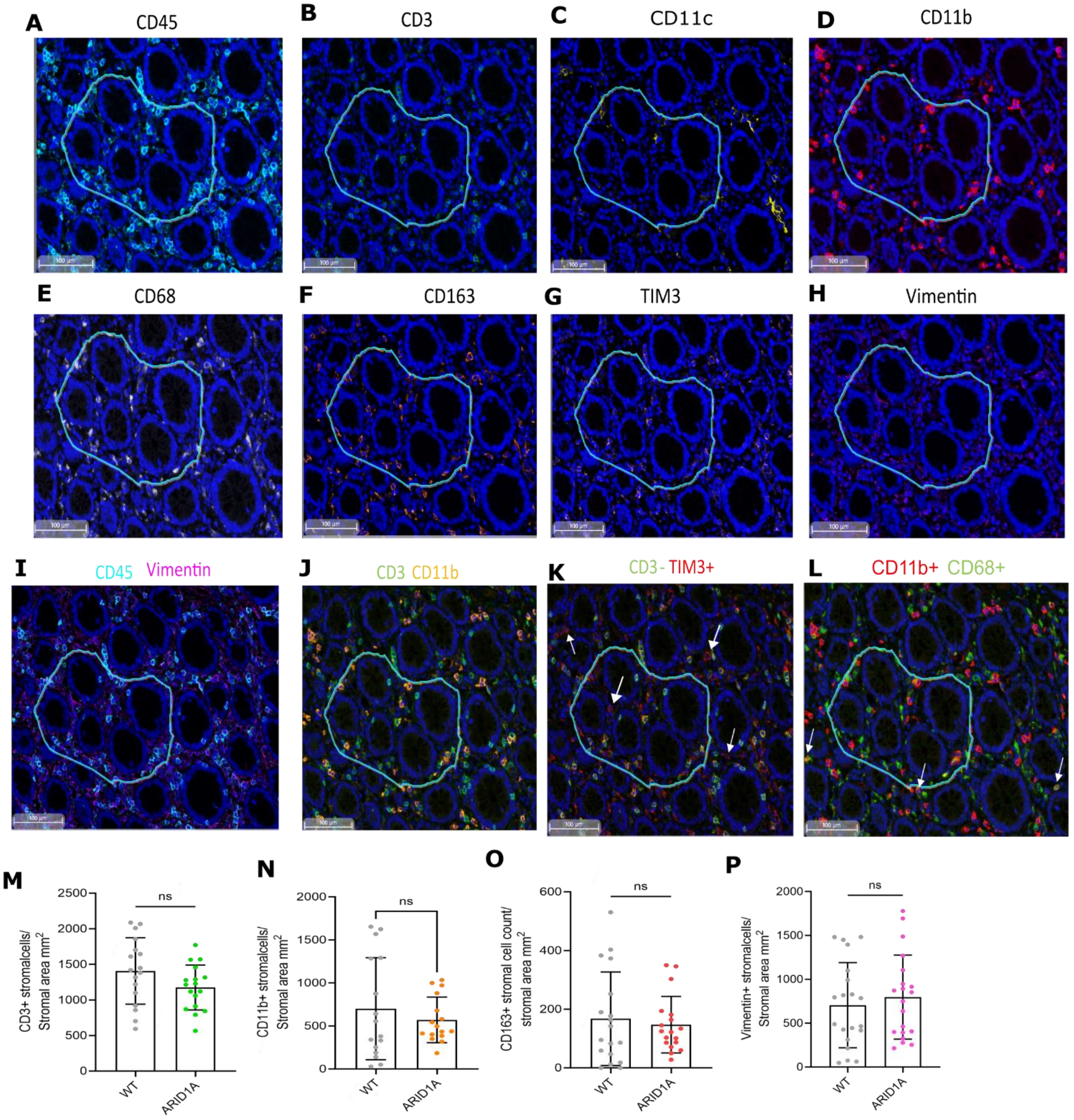

Appendix Figure S4: Signal star specialised stromal cell panel to profile cell populations within and around ARID1A patches. (A-H) Individual markers around an ARID1A patch shown in blue. Arranged in order of staining. (I) Separation of immune cells (CD45+) and mesenchymal cells (Vimentin+). (J) Separation of lymphocytic T-cells (CD3+) and myeloid cells (CD11b+). (K) TIM3+ is a marker of both T-cells and macrophages. TIM3+ cells that are CD3- are indicated by the arrows. (L) CD68+ cells are a sub cluster of CD11b+ cells (indicated by the arrows). (M-P) Quantification of stromal cells in WT ad ARID1A deficient patches. Showing non-significantly different stromal populations. (M) CD3+ cells. N= 17 ARID1A and WT clones. (N) CD11b+ cells. N= 16 ARID1A and WT clones. (O) CD163+ cells. N= 18 ARID1A and WT clones. (P) Vimentin+ cells. N= 20 ARUD1A and WT clones. Data information: Data presented as mean ± SD. Scale bars indicate 100 µm. Paired Wilcoxon test was performed to assess statistical significance. (M) $p = 0.0638$, (N) $p = 0.495$, (O) $p = 0.766$, (P) $p = 0.648$.

Appendix Table S1: Patient ages and gender.

Tissue Bank ID	*Age*	*Sex*
TB18.2900	*77*	*M*
TB18.3648	*72*	*M*
TB18.2315	*61*	*F*
TB18.3985	*81*	*M*
TB18.3753	*56*	*M*
TB18.3919	*70*	*M*
TB18.4180	*75*	*F*
TB18.3127	*71*	*F*
TB18.2870	*57*	*M*
TB18.3062	*71*	*M*
TB18.2959	*65*	*M*
TB18.2766	*84*	*M*
TB18.1979	*83*	*M*
TB18.1314	*59*	*F*
TB18.1270	*89*	*M*
TB18.2984	*79*	*M*
TB18.3071	*74*	*F*
TB18.4183	*76*	*F*
TB18.4560	*87*	*M*
TB15.4012	*78*	*F*
TB16.0049	*85*	*M*
TB16.0126	*72*	*F*
TB18.0210	*80*	*M*
TB18.1020	*79*	*F*
TB18.0068	*85*	*M*
TB17.2797	*70*	*F*
TB16.2117	*72*	*F*
TB17.1198	*30*	*M*
TB17.1215	*36*	*M*
TB16.0622	*37*	*M*
TB16.1540	*82*	*M*
TB16.0121	*72*	*F*
TB18.2520	*71*	*M*
TB18.2156	*79*	*F*
TB18.1962	*38*	*F*
TB18.0219	*48*	*F*
TB18.0459	*48*	*F*
TB18.1997	*52*	*M*
TB16.0623	*70*	*M*
TB18.3910	*67*	*F*
TB18.4571	*60*	*F*
TB13.0420	*78*	*M*
TB12.2355	*77*	*F*
TB12.1904	*79*	*F*
TB12.1256	*81*	*M*
TB12.0017	*64*	*M*

TB11.1545	*79*	*M*
TB18.4245	*84*	*M*
TB19.1770	*57*	*M*
TB19.02072	*62*	*M*
TB19.02084	*62*	*F*
TB19.02261	*69*	*M*
TB19.02388	*79*	*F*
TB19.1496	*41*	*F*
TB19.1497	*62*	*F*
TB19.1589	*80*	*F*
TB19.1166	*68*	*F*
TB19.1674	*61*	*F*
TB19.1682	*57*	*F*
TB18.4761	*70*	*M*
TB18.5023	*62*	*F*
TB18.3673	*75*	*F*
TB18.4562	*59*	*F*
TB18.5947	*79*	*M*
TB18.5484	*78*	*M*
TB18.4864	*74*	*M*
TB18.5487	*42*	*M*
TB18.5262	*33*	*M*
TB18.4167	*76*	*F*
TB18.4844	*86*	*F*
TB18.4797	*87*	*M*
TB18.5068	*65*	*F*
TB19.0995	*39*	*F*
TB19.0996	*78*	*M*
TB18.3920	*58*	*F*
TB19.0997	*38*	*M*
TB19.0994	*62*	*M*
TB18.4121	*47*	*F*
TB19.1748	*62*	*M*
TB19.1721	*50*	*F*
DW20.1126	*74*	*F*
DW20.1122	*43*	*M*
DW20.1121	*83*	*F*
DW20.1130	*78*	*M*
DW21.1238	*49*	*F*
DW20.0901	*62*	*M*
DW20.1234	*67*	*F*
DW20.1235	*72*	*F*
DW20.1231	*54*	*F*
DW20.1125	*56*	*F*
TB20.01933	*40*	*F*
TB20.01912	*71*	*F*
DW20.0905	*87*	*F*
DW20.0904	*61*	*F*
TB17.1858	*83*	*M*
DW20.0907	*71*	*F*
TB17.2518	*40*	*M*

DW20.1129	*81*	*F*
TB20.01932	*72*	*F*
TB21.00044	*89*	*M*
TB20.01965	*59*	*F*
TB18.2700	*39*	*F*
TB17.1172	*46*	*F*
TB17.1274	*64*	*M*
TB17.1446	*80*	*F*
TB17.1449	*76*	*F*
TB17.2171	*48*	*F*
TB18.0493	*34*	*M*
TB18.1667	*63*	*M*
318_85	*56*	*F*
1130_85	*58*	*F*
TB16.0229	*48*	*M*
TB16.1164	*77*	*F*
TB17.1469	*75*	*F*
TB17.1465	*72*	*M*
TB17.1476	*84*	*F*
TB17.1512	*65*	*M*
TB17.2235	*82*	*F*
TB18.0650	*80*	*M*
TB15.1120	*55*	*F*
983_85	*66*	*F*
TB17.1522	*69*	*M*
TB18.0626	*72*	*M*
TB15.3375	*85*	*F*
TB18.0630	*35*	*M*
TB16.0052	*69*	*M*
TB17.2294	*80*	*M*
TB15.0995	*70*	*F*
TB16.0273	*56*	*F*
TB16.1125	*74*	*F*
TB17.2190	*78*	*F*
TB18.0342	*72*	*M*
TB15.1196	*64*	*F*
472_85	*84*	*F*
1092_85	*82*	*M*
5900_85	*65*	*F/M*
TB15.4025	*89*	*M*
TB17.1490	*76*	*F*
TB18.5138	*72*	*F*
TB18.5543	*65*	*F*
TB18.5801	*79*	*M*
19B_R_032	*64*	*M*
TB18.5961	*64*	*M*
TB18.5880	*81*	*M*
TB18.5823	*66*	*M*
TB19.0223	*81*	*M*
TB18.5704	*57*	*F*
TB19.1108	*75*	*M*

TB19.1064	*75*	*M*
TB21.00351	*76*	*M*
TB21.00227	*68*	*M*
TB21.00228	*71*	*M*
TB21.00340	*72*	*F*
TB21.00343	*71*	*M*
TB21.00342	*83*	*M*
TB21.00201	*66*	*F*
TB21.00180	*75*	*F*
TB21.00398	*74*	*M*
TB18.2657	*75*	*M*
TB16.0059	*49*	*F*
10464_85	*68*	*F*
TB16.0624	*68*	*F*
TB16.0382	*83*	*F*
TB16.0431	*83*	*F*
TB16.1083	*74*	*M*
TB16.2167	*68*	*F*
TB16.0136	*77*	*F*
TB17.1245	*77*	*M*
TB17.1508	*71*	*M*
TB17.1538	*82*	*M*
TB17.1879	*57*	*M*
TB17.2722	*61*	*M*
TB17.1862	*61*	*F*
TB11.1767	*91*	*F*
TB17.2426	*80*	*M*
TB18.1283	*79*	*F*
TB17.1524	*64*	*M*
TB17.1292	*67*	*F*
13S 09820 AB	*60*	*M*
13S 08692 B	*42*	*M*
TB15.4011	*80*	*F*

Appendix Table S2: Multiplex IF panels.

**Panel name**	**Targets order**	**Fluorophore order**	**Multiplexing method**
Clonal Marks-1	SMAD4 PTEN STAG2	Cy5, Cy3, 488	Standard TSA
Clonal Marls-2	ARID1A KDM6A MAOA	Cy5, Cy3, 488	Standard TSA
Epithelial clonal properties lineage	MUC2 MCM2 ChgA	Cy5, Cy3, 488	Standard TSA
Epithelial clonal properties cell cycle	ANLN CA2+pH3	Cy5, Cy3 + 488	Standard TSA
General Immune cells	CD8 NE CD4 CD20 CD68 Ecadherin	480, 520, 570, 620, 690, 780	Opal TSA
Specialized Immune cells	Round 1: CD45 CD3 CD11c CD11b<br>Round 2: CD68 CD168 TIM3 Vimentin	488, 594, 647, 750	Signal Star

Appendix Table S3: Fluidigm Primers for PTEN exon coverage used for Juno chip and pooling strategy. Lower case letters indicate intron binding.

Amplicon	Sequence F' primer	Sequence R' primer	Product Size	Multiplex Group
**TXA0022214**	TTTTCTTTCTCTAGGTGAAGCTGT	TTCATGGTGTTTTATCCCTCTTGA	235	MP1
**TXA0429431**	CCACAGTTGCACAATATCCTTTT	AAAAATTTGCCCCGATGTAATAA	190	MP1
**TXA0343717**	TCTGTCTTTTGGTTTTTCTTGAT	GTTGTTTTAGAAGATATTTGCAAGC	190	MP1
**TXA0394520**	TATTGCCCCTTAAGACCTTCCAG	ACAAAAACATATTACACAGCTACACA	219	MP1
**TXA0001811**	AACATAGGTGACAGATTTTCTTTT	GCTCTATACTGCAAATGCTATCG	217	MP1
**TXA0052316**	GACCCGGGCCGGTTTTA	GCCGCTTGGCTCTGGAC	229	MP1
**TXA0342048**	GTCAGAGGCGCTATGTGTATTATT	TCCTGCATAAATTTCAAATGTGGTAA	240	MP1
**TXA0018633**	TCACTGTAAAGCTGGAAAGGGAC	TCAGATCCAGGAAGAGGAAAGGA	172	MP2
**TXA0429393**	GCTGCAACCATCCAGCAG	CTGTGGCTGAAGAAAAAGGAG	177	MP2
**TXA0001785**	AGCTCATTTTTGTTAATGGTGGCTT	ACTCTACCTCACTCTAACAAGCAGA	184	MP2
**TXA0429400**	GCTACCTGTTAAAGAATCATCTGG	ACTTCTAGATATGGTTAAGAAAACTG	163	MP2
**TXA0342046**	TCCAAACATTATTGCTATGGGATTTC	TCACAAAGTATCTTTTTCTGTGGCTT	180	MP2
**TXA0429383**	CCAATTCAGGACCCACACGA	AAACACCTGCAGATCTAATAGAAAAC	240	MP2
**TXA0429384**	TCAAGATTGCAGATACAGAATCCA	ATGAACTTGTCTTCCCGTCGT	211	MP3
**TXA0005243**	GTGTCACATTATAAAGATTCAGGCAA	ACAGTAAGATACAGTCTATCGGGTT	210	MP3
**TXA0116015**	CAAATCCAGAGGCTAGCAGT	AAAGGTCCATTTTCAGTTTATTCA	216	MP3
**TXA0032268**	GGACCAGAGGAAACCTCAGAAAA	ACACACATCACATACATACAAGTCA	240	MP3
**TXA0346890**	GAACGCCGGAGAGTTGG	CGGAATGGGGAGAAGACG	189	MP3
**TXA0342045**	AGGAGAAGCAGGCCCAGTC	CGATCTCTTTGATGATGGCTGTC	231	MP4
**TXA0018629**	TCTGTCCACCAGGGAGTAACTAT	TGGAAGGATGAGAATTTCAAGCACT	182	MP4
**TXA0429386**	AGTAGAGTTCTTCCACAAACAGAAC	TCACCAATGCCAGAGTAAGCAAA	179	MP4
**TXA0149228**	GATATTCTGACACCACTGACTCT	CAAGATTGGTCAGGAAAAGAGAA	222	MP4
**TXA0061549**	AGCGTGCAGATAATGACAAGGAA	GCTGTACTCCTAGAATTAAACACACA	183	MP4
**TXA0343718**	TCTTCCTAAGTGCAAAAGATAAC	TCGATAATCTGGATGACTCATTATT	189	MP4
**TXA0429390**	AGGGAGGGGGTCTGAGT	GAGAAGACGAATAATCCTCCGAA	222	MP5
**TXA0030487**	TGACCACCTTTTATTACTCCAGCTA	AGCATTCTTACCTTACTACATCATCA	228	MP5
**TXA0115976**	TGAGGTTATCTTTTTACCACAGTTGC	TGCACATATCATTACACCAGTTCGT	180	MP5
**TXA0429387**	AAAGATCATGTTTGTTACAGTGCTT	TGAACTGCTAGCCTCTGGATTTG	202	MP5
**TXA0001787**	ATGGCTACGACCCAGTTACCATA	TTCCGCCACTGAACATTGGAATA	230	MP5
**TXA0429391**	CAGCCGTTCGGAGGATTATT	ACTTGGCGGTAGCTGATG	182	MP6
**TXA0429399**	TGTTCTTAAATGGCTACGACCCAG	TCCAGATGATTCTTTAACAGGTAGC	171	MP6
**TXA0429432**	TGCAACATTTCTAAAGTTACCTACT	CGTCCCTTTCCAGCTTTACA	220	MP6
**TXA0001779**	TCTTTTAGTTTGATTGCTGCATATTT	TTTCTAAATGAAAACACAACATGAAT	211	MP6
**TXA0343730**	GATAGTTTATTTTGTTGACTTTTTGC	TCACATAGACTTCCATTTTCTACT	219	MP6
**TXA0031783**	TCGTTTTTGACAGTTTGACAGTT	AGCATCTTGTTCTGTTTGTGGAA	230	MP6
**TXA0429389**	ACAAAATGTTTCACTTTTGGGTAA	TGCTTTGTCAAGATCATTTTTTGT	182	MP7
**TXA0429392**	GAAGAAGCCCCGCCACC	GTGACAGAAAGGTAAAGAGGAGCA	190	MP7
**TXA0031393**	TTTTAGCATGTGCTTCTGCTTATT	ACATACTTTAGCCTTGGCCTCTAC	219	MP7
**TXA0429394**	CCTCCTCTTCGTCTTTTCTAACC	CCCAGCCCTGGAAATGG	220	MP7
**TXA0429385**	GTCTGCCAGCTAAAGGTGAAGAT	TCCCAATGAAAGTAAAGTACAAACCT	177	MP8
**TXA0342047**	CAATCATGTTGCAGCAATTCAC	AGAAACCCAAAATCTGTTTTCCA	221	MP8
**TXA0129573**	CGTCTTTTCTAACCGTGCAG	AGAAGGGGAGAGACCAACTC	240	MP8
**TXA0377194**	aTTTCCATCCTGCAGAAGAAGCC	CCCACGTTCTAAGAGAGTGACAG	220	MP8
**TXA0429388**	AGATGAGTCATATTTGTGGGTTTTCA	TCTTCATCAAAAGGTTCATTCTCTGG	205	MP8
**TXA0429398**	TGTTACTAGTTTACGTGTTTACAG	CACTGTCTTCCACCTATACATCT	207	MP8

Appendix Table S4: Fluidigm Primers for ARID1A exon coverage used for Juno chip and pooling strategy. Lower case letters indicate intron binding.

Amplicon	Sequence F' primer	Sequence R' primer	Product Size	Multiplex Group
**TXA0429468**	GGGCGTAATGACATGACCTATAA	GGGGCAGAGGGACCATA	182	MP1
**TXA0429470**	AGAGTAGCTTCACTGATGGG	GCAGGATGTTGATGGTATCTAATG	176	MP1
**TXA0068511**	AGTAAGCCTGCCTGGTTTATCAAT	ATTGGTTTCCTCTCTGCCCCTAT	229	MP1
**TXA0068558**	ATGGATCAGATGGGCAAGATGAG	ACAGCAACAAGGGTCAAGGTAAT	240	MP1
**TXA0429480**	GAGAGCATTTGCCTGCCTGTC	GGGGTGTGGCCAGAATCA	187	MP1
**TXA0429438**	AACCAGCAAAGTCCTCACCC	CCGGGGACTGCTGATGTG	181	MP1
**TXA0429481**	CAGGGCTGCTGCTCATCC	CGAGTGTAACCAAGGTGTTTTCC	180	MP1
**TXA0429487**	CCTCAGTGACCGAAAGAACCC	ACTAGTTGGCTCAAAGGGTGG	220	MP1
**TXA0013937**	CAACAACATGGCGGACAACAAAG	GGAGCTCAGCGCGTAGG	189	MP1
**TXA0429479**	AGATCTTTGGCATTTTAAAGGAGT	CTGAAGCTGGCTTGTCCTTG	219	MP1
**TXA0429489**	CCGAGGATGGAGCTAAGAGTT	GCACAAATGACAGGCTTCG	216	MP1
**TXA0429450**	ACAGCTAAACTTACTGGACTTGA	TCCTTCCTATCACTGAAAAAGAT	217	MP1
**TXA0001721**	AGGAAAATGCTAAGCAAGTAGTAGG	CAGGTGAGGGGAGGTATGA	236	MP1
**TXA0429448**	GGCATGGCTGGAGGCATAAA	GACAGCAGTTTCTTGGGTTTTCC	219	MP1
**TXA0068523**	TGCTGCCAGCTCCTTGAAAA	CATATCCTGAATAAGAGGCCAGGG	216	MP1
**TXA0378755**	GGTCTCGGTGCTGCTATGGAT	TAGTTTTCAAGGCGAACCTGCAT	220	MP1
**TXA0068560**	AAAGTCCCAGGATAAGGATGGAG	ATAGCTCCCCATGGAGTTCTGC	216	MP1
**TXA0001746**	GTGAGCACATCAGGGATTTCCA	AGTCACCTTTCCCTCTCCCTAAA	223	MP2
**TXA0386811**	GGGCCGAGAGCAATGGG	GTAGGGTTGCCCGAAGCC	236	MP2
**TXA0001728**	GCAATGCCTATCCTGCCACT	TAGGTCATGTCATTACGCCCCTG	223	MP2
**TXA0068507**	GGGCCAGACTCCATATTACAACC	CGACTGCTGGGAGGGGTAT	223	MP2
**TXA0068559**	CAACCTGGGCTTGGTGGATAG	AAGATCCCAAACCCTCTCAATCT	218	MP2
**TXA0429441**	TATCAATACCAGGCCATCACAGC	ACCTTTCAGAAGGTGCAGAAATA	188	MP2
**TXA0068565**	CATGGCGTGAACCGAACAGATG	GAGAGGTGCGGTTCTCCATT	232	MP2
**TXA0429440**	GAACTCCCCAGCCCACC	CTTTGTTGGGCCCCTCCC	214	MP2
**TXA0429453**	TGTGGGCACATCAAGCAGT	GGCCGCTGACCCCATC	185	MP2
**TXA0429458**	TGGGTCAAAGGGTAGATTACCAG	GTCCCATCGCCACATTTCCTA	178	MP2
**TXA0068517**	AGGGATTTCTTCAAGAGTCACATCA	AGGACACAAAGTTGAAAGGAGTCA	218	MP2
**TXA0429483**	AGGTCCTAAACTAGAAGAGGAAG	CAAGCTTATCTGAGCAGTCCA	188	MP2
**TXA0429485**	CCACACAGTTCCAGCAGAG	GTGATACCGAGATGTCCAACAG	180	MP2
**TXA0429477**	GAACCCCACAGTAAGGATGAG	CTTGTGGTGCAGCAGGAT	189	MP2
**TXA0001760**	TATATGCAGAGGAACCCCCAGAT	ACTGTTTTCTCCTCTCACCCGTA	217	MP2
**TXA0429486**	GTTCTTAGGCCACTTTTCTCCCT	GACACCTTGCTGAACCTCCCA	190	MP3
**TXA0429434**	GCAGCAAGGACATGGGT	CTCACAGATCAGATTTTGGACAG	176	MP3
**TXA0378744**	AGGCTACCCCAATATGAATCAAGG	GCTGCCATCCCTGTAAAAGAGAAA	240	MP3
**TXA0211687**	CAACGGCGGGATGGGTG	GCAGATTGAGCCCACTATAGCTT	176	MP3
**TXA0429471**	AATGATGTCCCTCAAGTCTGGT	TGGCTAAAGATGAGACATTCCC	190	MP3
**TXA0068544**	CATCCCACCTTATGGCACACTC	CTTGCACTGACACCCTCTCT	231	MP3
**TXA0068557**	GGCCTCTTCATGAGCCATTTCTA	CTCAAATGTCTGCCCTAGCTCC	239	MP3
**TXA0068561**	GTCCTCTCAGCCTCCATACTCC	GCAGTTTGCTGGGACTGCT	237	MP3
**TXA0068514**	CAGCAGAACTCTCACGACCAC	CCCCATGGCCCCGAAG	231	MP3
**TXA0429463**	CACCCCCTGGCACCAAT	CTCTGATCTGTGTGCAGCATTTC	217	MP3
**TXA0068531**	GACTTCTGAGACCCTTAGCACAG	TAGAGGTCCAGAGGTTTCCTACC	237	MP3

**TXA0429488**	TGACAACAGCAGAGGGTACA	GGGCTAATGCCAAATGGAAAC	190	MP3
**TXA0068546**	GAAGTGACTCCACATTCCAGAAG	CTGAGCCATTCTCAAGATTTCC	217	MP3
**TXA0068564**	GGAAAACCAGGCGGGAGATATAC	CTGGGACTTCTTGGAATCAGCA	233	MP3
**TXA0429482**	TCTTGGAAACCCTCAGCAAACTC	GCTGTCCCCCTGAGCC	185	MP3
**TXA0386804**	ATGGCCTCGCAGTGTT	ACTGAGGGGAGCCCAA	240	MP4
**TXA0378770**	GATGACATGTTGTCTACTCGGT	CTTCGAATGGTATTGGACACAC	236	MP4
**TXA0386808**	TGAAGCCCAGGACCCCTTTT	CAGCAGTACCACAGCCATCTC	223	MP4
**TXA0429473**	TTTTAAAGGAGTATGAGGTGGGT	GATCAGCTTCTCCTCACTATTCT	230	MP4
**TXA0001737**	GGAGATGTACAGCGTGCCATA	GCCAAACTGGAATGGAAATTGGT	223	MP4
**TXA0001752**	CAAGGCCCCCTCCATCTAACTA	GTGATATCCCGCCGAATCATGG	226	MP4
**TXA0001765**	GTGAGCCTGAGAGGAAGATGTG	AGCCAGTGAGTACCTAGAAAGGG	221	MP4
**TXA0429444**	TCCTAGTTAGAATTCCCAGGCTT	CCCCTCCCCAACACCCA	177	MP4
**TXA0429447**	ATGGGCAACCGGCCTTATG	GCATCTCAATAATCTGAGTGGGTCT	219	MP4
**TXA0013935**	TGATGGGAACTGGACCTCCTTAT	TTTAGCTGTGATGTGACTCTTGA	166	MP4
**TXA0429454**	GATGGGGCTTGGGGCTTATG	TCTCCCCGTTCAATCTTGCATTC	208	MP4
**TXA0429456**	GACTCCTGCGTGTCCTTTGTTAT	CAGAGGTATTCATACTGGGCTGA	190	MP4
**TXA0429459**	CTGCCCACCCTAATCCTG	AGACTTCAACACGTTAGATTAGTT	218	MP4
**TXA0068556**	TCATCAGTGCATAGCTTCTCACA	GATACGAAGGTTGGGCATGAGG	237	MP4
**TXA0429451**	AGAGGAAGATGTGGGTGGAC	GAAACCACAGGGGCAGTCAAG	182	MP5
**TXA0001747**	GGCTTGTCAACTTACCAGTTTGTTC	GGACAGGAGCTGGGGTCAT	224	MP5
**TXA0429455**	TGTGAGAGTTAAACACTGTCATGC	CATCTGCCCCTGCTCTTGG	217	MP5
**TXA0429472**	AGAAGAATGATCCATTTGTGGTG	TTGTCCCTGGTGTACCCT	222	MP5
**TXA0386815**	GGCTTCGGGCAACCCT	TAGGCGCTGCGGTTGG	221	MP5
**TXA0001734**	TGTTTGGTGTTCTAGAGTTGAGAG	GAGTGTGCCATAAGGTGGGATG	220	MP5
**TXA0429443**	CTCATACCTCCCCTCACCT	TGGAAGACTAGGGCTCCTT	190	MP5
**TXA0429433**	GGGTTATATATTCAGTGGCCAGAGG	GCTGCGGGGTCTGGGA	214	MP5
**TXA0429437**	CCACAGCTCCAGTCCTCTCA	GTGCTGGCTGCTGAGGT	184	MP5
**TXA0378765**	CTTCCTAGAGGACAGCCTTG	ACAAATGACTTGTGAAACCAATGA	238	MP5
**TXA0378771**	GACTTTGAGATGTCCAAACACC	GGTATGGAGATAGGTCCAACTG	238	MP5
**TXA0429465**	AAATGCAGAAGGCAGGTCCC	TTAGGCAACCGAATGAGGAAGAT	190	MP5
**TXA0429469**	CCTTCCCCTCAGCAAGATGTAT	CAGCTGATGCCTGTATGGG	208	MP5
**TXA0013973**	GATGGAGAGCATTTGTTCGCATT	CCCATGCCTGTGTGTATCTGTC	169	MP6
**TXA0386806**	ccctcaaccAACTGCTCA	CTCCCGGGGCTCATGG	217	MP6
**TXA0068535**	CAAACTATAATGCCTTGCCCAAT	GACACATCCCTGACCCAAC	216	MP6
**TXA0429478**	AAGGTGGACGAGAACCAC	GTTTCTAAGTTCTCCACACACG	190	MP6
**TXA0068566**	CACTGTCATGCCAAGCAAACTAC	CCCATGCACTTATCTTCAGCCAT	236	MP6
**TXA0429452**	CCCTTAGCACAGGCTTTGAATCT	GTCATGCCCATGGCCTTCT	187	MP6
**TXA0429484**	TGAGCTGCAACAAAGTGGAGT	GGGAAAGGACGGCATTGGG	219	MP6
**TXA0001733**	CAGAGGCCATCAAAGCTCAGG	CCCTGCATGGTCATCGGGTA	217	MP6
**TXA0429466**	CCACCAAGCATGCAGAATCACA	TTCAACAGAGCCAGGTGGGAA	219	MP6
**TXA0001708**	GCAAAACATGCCACCACAAATGA	CCATGGGAAGGCCACGAG	228	MP6
**TXA0429460**	ACAGAGTGAGGTAAGCATGAC	AGGAGAATACATCCCCGAGT	190	MP6
**TXA0429436**	CACAGCCACAGGCTCAGT	TAGTCACACACAGGGAAGGG	190	MP6
**TXA0429475**	GGGACACCACTGAGCAT	ATAGTGGCTGTGATCCGTTTTTC	187	MP6
**TXA0068506**	TTTATGTCCCTGAGTGCAGAGTA	GTATACATCTTGCTGAGGGGAAGG	237	MP6
**TXA0378761**	CATCAAGATCCTAGAGGACGAAC	TAAGTTAGTGGTGCCTGCTTC	240	MP7
**TXA0429462**	GGCACGAAGGGGAGATGTA	CGGCGCTCAGTAGCAG	190	MP7
**TXA0386814**	GCAGCAGCGGGAGGAG	GTTCCCGTTCGAGTTCTTCAGG	232	MP7
**TXA0429442**	GCTCTTGGTTGTTTAAGGAAAATGC	GCTGGATTACTCTGCTCTCCTTG	214	MP7

**TXA0429446**	AGTTGAGAGATATTAGTGAGTTGCT	CTGTCCATGCATTTGACCTC	178	MP7
**TXA0429457**	TCCAGGATGCCTTTAATGATGGA	AGCACAAGTTCAAATAGCAATCAG	189	MP7
**TXA0378769**	GCCGCCTGGAGAAGTTGTAT	GGTTCTGCATGTGGAGGAGG	237	MP7
**TXA0386817**	ACCCTGAAGCTATAGTGGGCTC	ATCCATAGCAGCACCGAGA	219	MP7
**TXA0378772**	GACAGAGCTGCTGCCTT	GAAACTTGCTGCTCTCCTTGAT	233	MP7
**TXA0001730**	TTCCTGCACTCTGGGATGAAAAT	AAGATGGAAAGGGGCAGATTAGG	227	MP7
**TXA0386807**	TGGGGAGGTCTCTCAAGTCAATA	CCCTCCATGGGAGCTGGAC	239	MP7
**TXA0429435**	TCATGCAGGAGAGTCAGTGCTAA	GGTCTGGGACGGTGGTTG	175	MP7
**TXA0429449**	ACTAGATGATCACACAGCACTATTT	GGTTGATCATGCCAGCCATAC	189	MP7
**TXA0429474**	tgatgaggaGATAGCCTTTTCAG	GTCTGGATATGCTCAGTGGTG	219	MP7
**TXA0068534**	ACAGCACTATTTGGCTCCAGTTC	GCACTTACCTGCAGAATTGTTGG	235	MP8
**TXA0429439**	CCGCCGGGCAGGAAAG	GGCTCCGTGAGGTTATTGTTCA	202	MP8
**TXA0429476**	AGTTTGACAAGCTTCCAGTAAAG	TTCCGAGGGGCTGGTG	217	MP8
**TXA0378766**	CTTGGAGATGCTCCGGGAAA	ATTGTTGTCCTGGATGCTGAGTT	238	MP8
**TXA0386810**	GCGCTGCTTGCCTTGG	GCATAAATAAAGGGCAACAGTCA	231	MP8
**TXA0001711**	CCCCCTACTCACAGCCACAG	GTTCTCATGACACTAACCCCCAA	230	MP8
**TXA0429467**	ATCTTGGCATCTGTGGGCTTTAT	AACTGCTGCTGCTGAGG	185	MP8
**TXA0429464**	CACAGATCAGAGGGCCAACC	CAGGAATGGAGACTTGCTAGGAG	220	MP8
**TXA0013972**	TTCAATAGATGACCTCCCCAT	CAACACTGGCGGGAGAG	188	MP8
**TXA0429445**	GATGCCCCAGTACAGTTCCC	CCCGTATCTACTCCTAACTGGGT	180	MP8
**TXA0068503**	TGGAGGAATTGGTTTATTTGTGGTT	ATCAGTCACCTTTCCTCATGCTG	234	MP8
**TXA0429461**	AGGGAAACATGAGCACTGG	CAAAGAAACACAGGATTAGGGTG	189	MP8
