# Supplementary figures and images for: Haploinsufficient phenotypes promote selection of PTEN and ARID1A-deficient clones in human colon
Source: EMBO Rep. 2025 Feb 7;26(5):1269–89. doi: 10.1038/s44319-025-00373-0 (PMC11893880; doi:10.1038/s44319-025-00373-0)

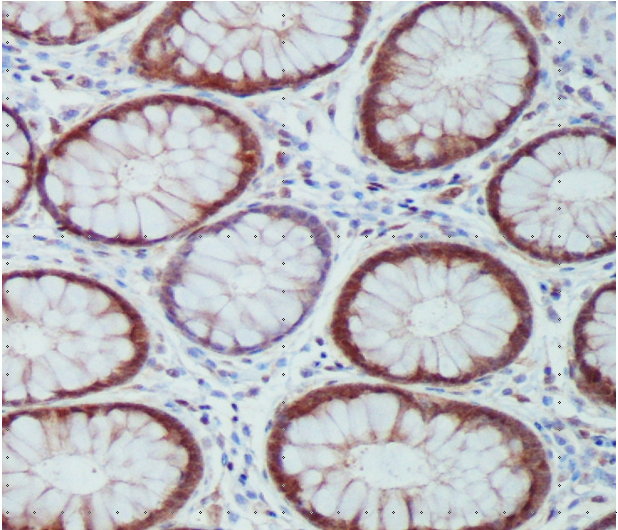

Supplement: Supplementary file 4 — Source data Fig. 2 [file 44319_2025_373_MOESM4_ESM.zip › New folder/2A-2F/2A-C images/2A.PNG]

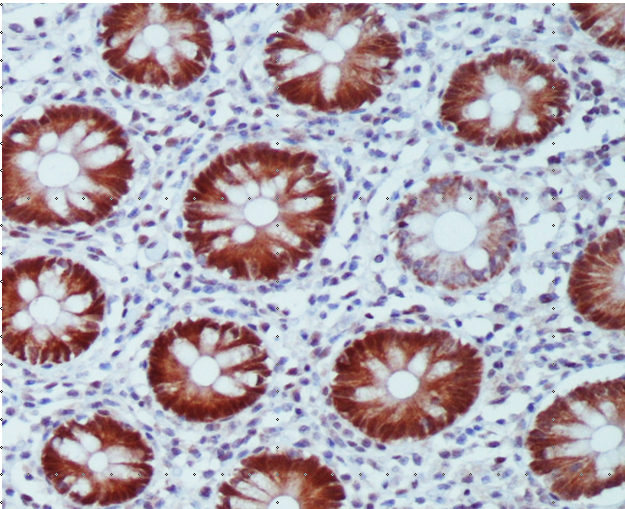

Supplement: Supplementary file 4 — Source data Fig. 2 [file 44319_2025_373_MOESM4_ESM.zip › New folder/2A-2F/2A-C images/2B.PNG]

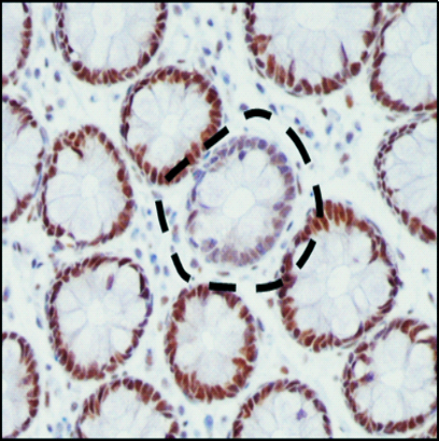

Supplement: Supplementary file 4 — Source data Fig. 2 [file 44319_2025_373_MOESM4_ESM.zip › New folder/2A-2F/2A-C images/2C.png]

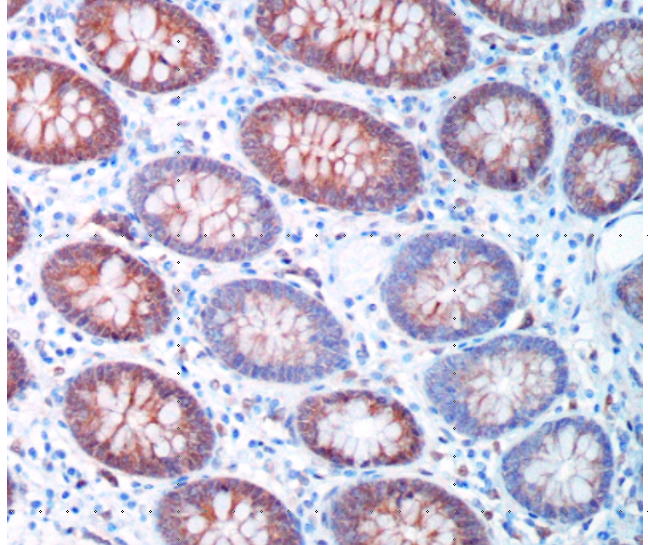

Supplement: Supplementary file 4 — Source data Fig. 2 [file 44319_2025_373_MOESM4_ESM.zip › New folder/2A-2F/2E images/2E_1.PNG]

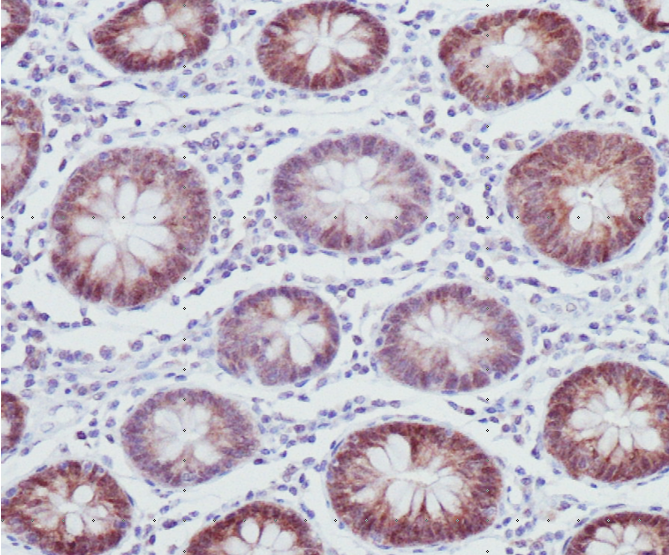

Supplement: Supplementary file 4 — Source data Fig. 2 [file 44319_2025_373_MOESM4_ESM.zip › New folder/2A-2F/2E images/2E_2.PNG]

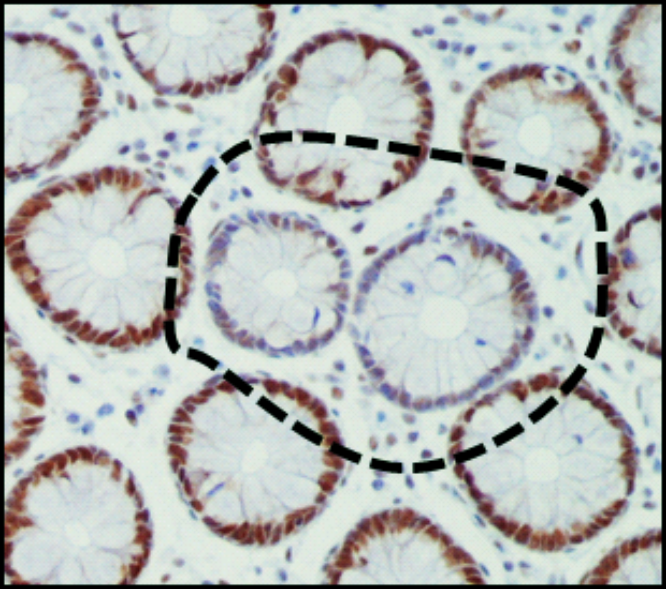

Supplement: Supplementary file 4 — Source data Fig. 2 [file 44319_2025_373_MOESM4_ESM.zip › New folder/2A-2F/2E images/2E_3.png]

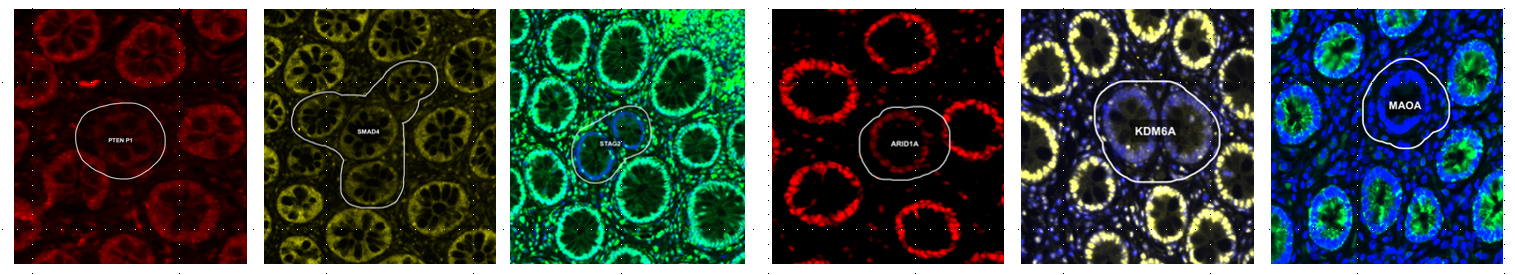

Supplement: Supplementary file 5 — Source data Fig. 3 [file 44319_2025_373_MOESM5_ESM.zip › Figure 3/3A/3A.PNG]

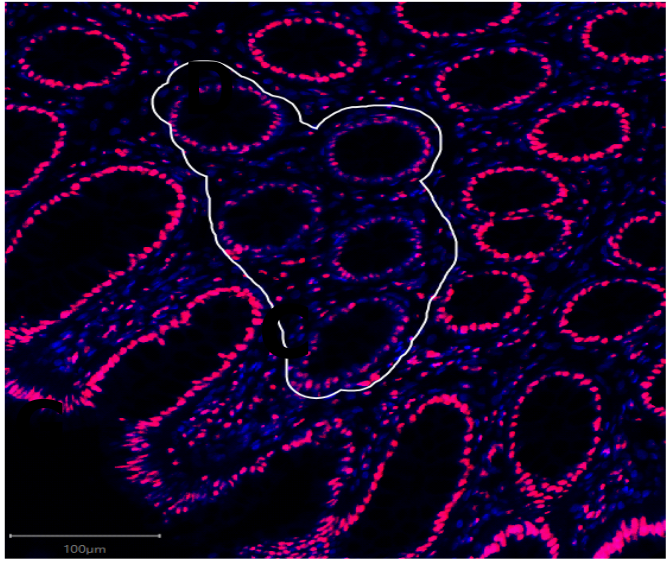

Supplement: Supplementary file 5 — Source data Fig. 3 [file 44319_2025_373_MOESM5_ESM.zip › Figure 3/3C/3C-ARID1A.PNG]

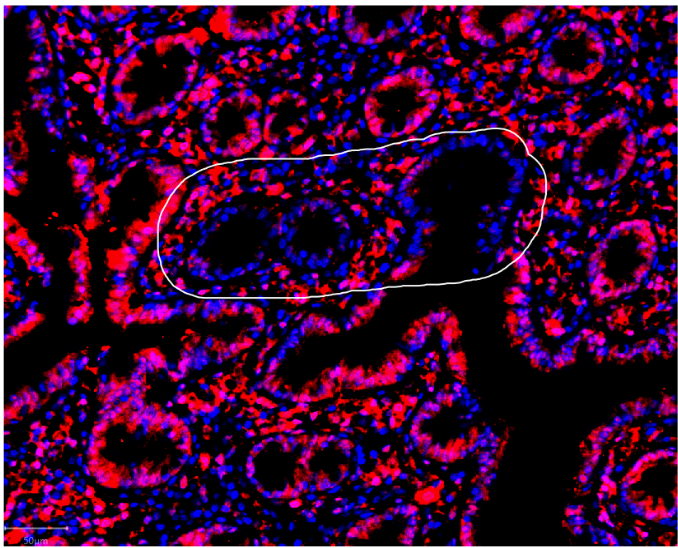

Supplement: Supplementary file 5 — Source data Fig. 3 [file 44319_2025_373_MOESM5_ESM.zip › Figure 3/3C/3C-PTEN.PNG]

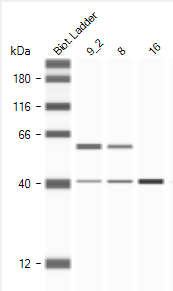

Supplement: Supplementary file 5 — Source data Fig. 3 [file 44319_2025_373_MOESM5_ESM.zip › Figure 3/3G-H/wes_snap_fig3g.PNG]

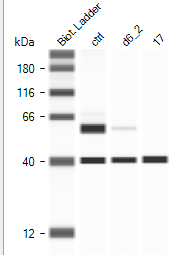

Supplement: Supplementary file 5 — Source data Fig. 3 [file 44319_2025_373_MOESM5_ESM.zip › Figure 3/3G-H/wes_snap_fig3g_2.PNG]

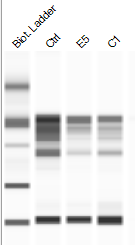

Supplement: Supplementary file 5 — Source data Fig. 3 [file 44319_2025_373_MOESM5_ESM.zip › Figure 3/3G-H/wes_snap_fig3h.PNG]

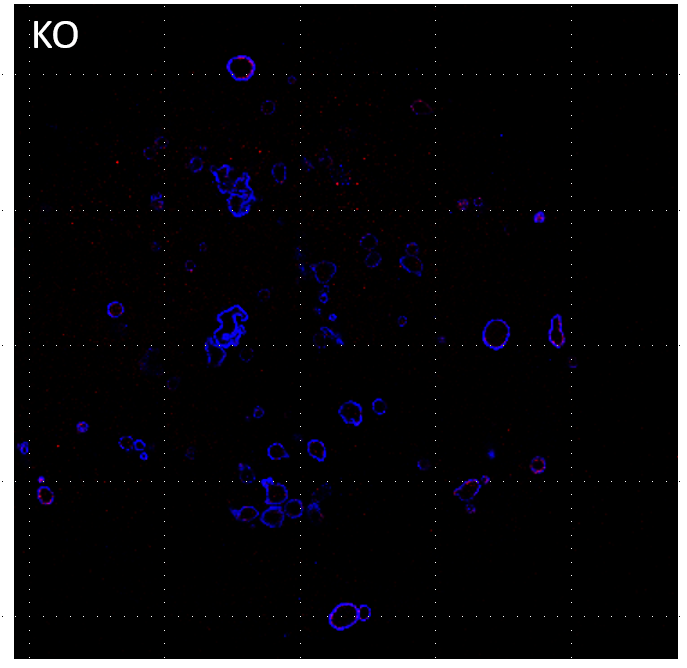

Supplement: Supplementary file 5 — Source data Fig. 3 [file 44319_2025_373_MOESM5_ESM.zip › Figure 3/3K-L/1_pten_ko.PNG]

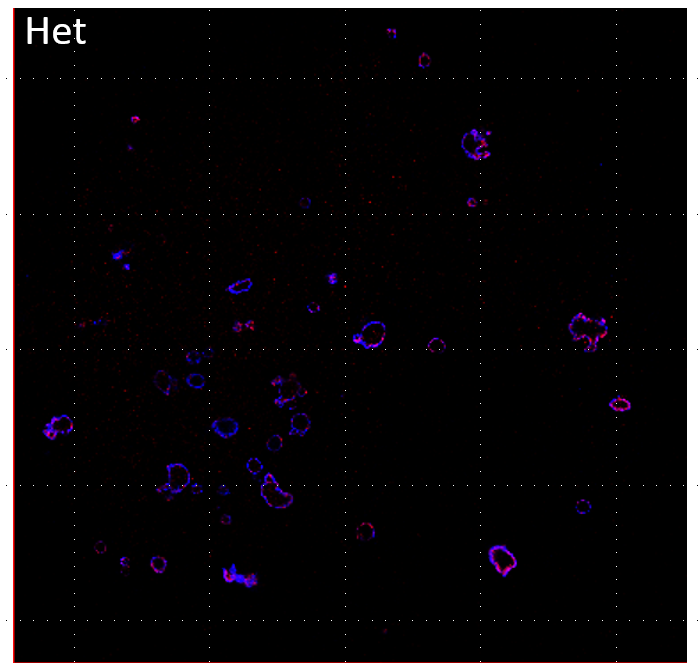

Supplement: Supplementary file 5 — Source data Fig. 3 [file 44319_2025_373_MOESM5_ESM.zip › Figure 3/3K-L/2_pten_het.PNG]

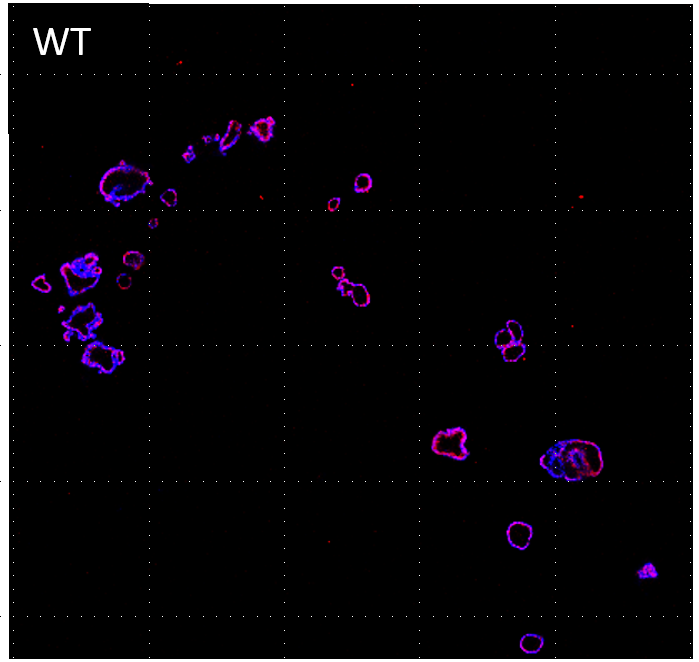

Supplement: Supplementary file 5 — Source data Fig. 3 [file 44319_2025_373_MOESM5_ESM.zip › Figure 3/3K-L/3_pten_wt.PNG]

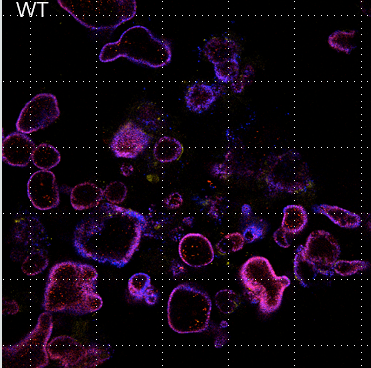

Supplement: Supplementary file 5 — Source data Fig. 3 [file 44319_2025_373_MOESM5_ESM.zip › Figure 3/3K-L/4_arid1a_wt.PNG]

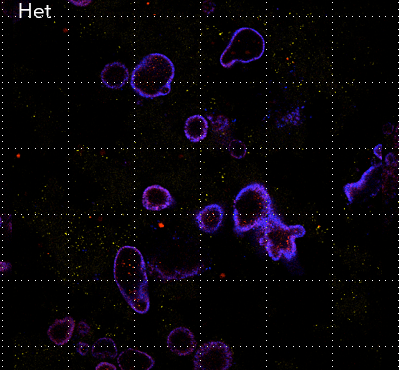

Supplement: Supplementary file 5 — Source data Fig. 3 [file 44319_2025_373_MOESM5_ESM.zip › Figure 3/3K-L/5_arid1a_het.PNG]

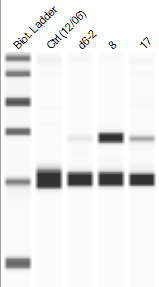

Supplement: Supplementary file 6 — Source data Fig. 4 [file 44319_2025_373_MOESM6_ESM.zip › Figure 4/4A-B/wes_snap_fig4a.PNG]

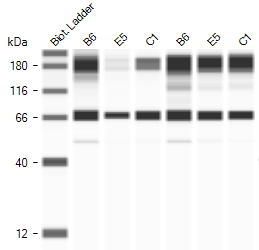

Supplement: Supplementary file 6 — Source data Fig. 4 [file 44319_2025_373_MOESM6_ESM.zip › Figure 4/4A-B/wes_snap_fig4b.PNG]

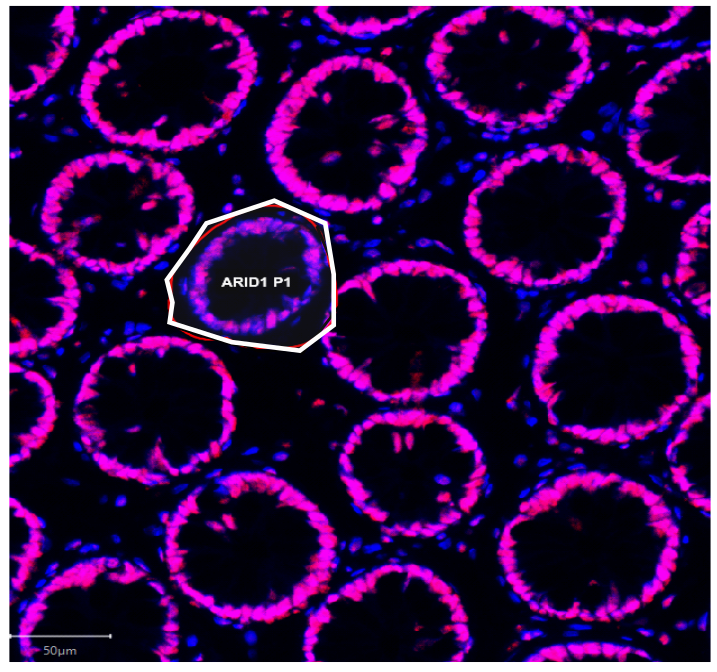

Supplement: Supplementary file 6 — Source data Fig. 4 [file 44319_2025_373_MOESM6_ESM.zip › Figure 4/4I/4I-1_ARID1A.PNG]

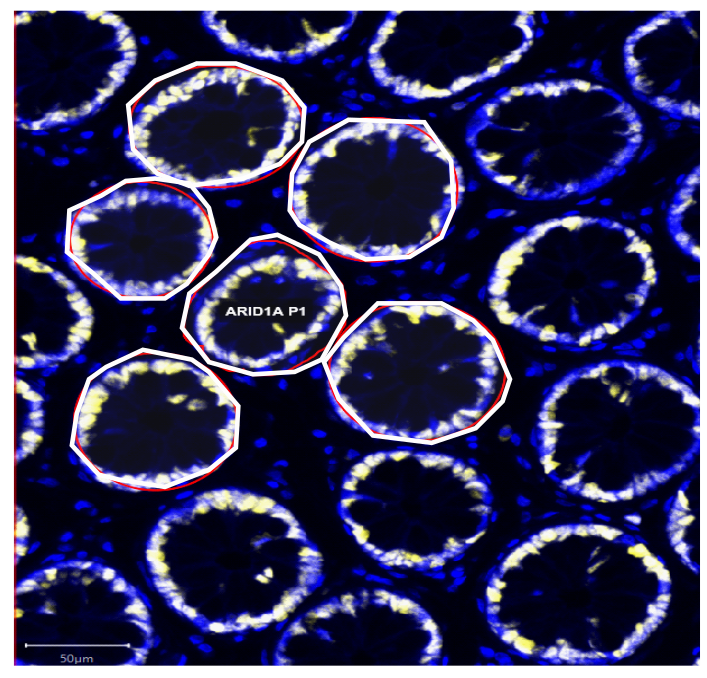

Supplement: Supplementary file 6 — Source data Fig. 4 [file 44319_2025_373_MOESM6_ESM.zip › Figure 4/4I/4I-2_MCM2.PNG]

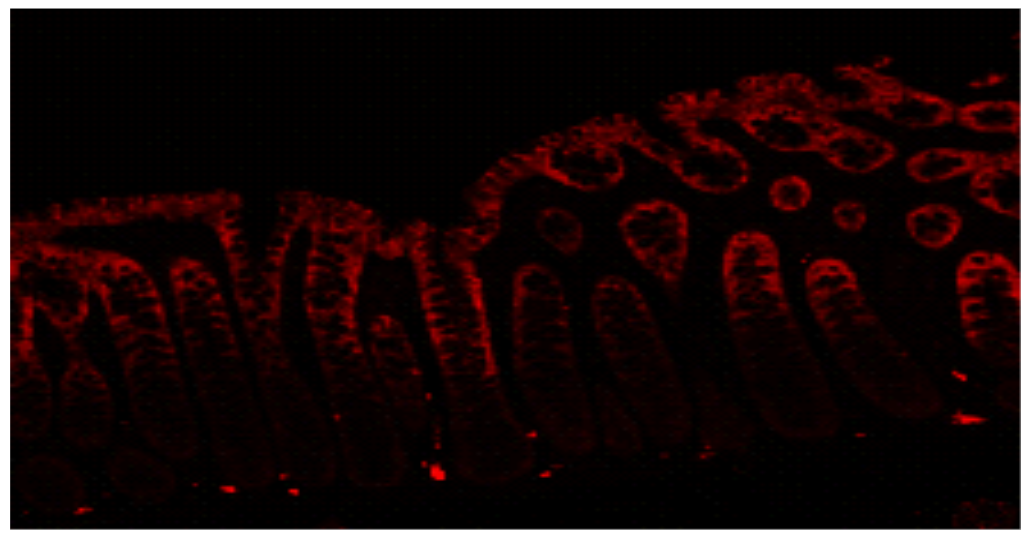

Supplement: Supplementary file 6 — Source data Fig. 4 [file 44319_2025_373_MOESM6_ESM.zip › Figure 4/4J/image_CA2.PNG]

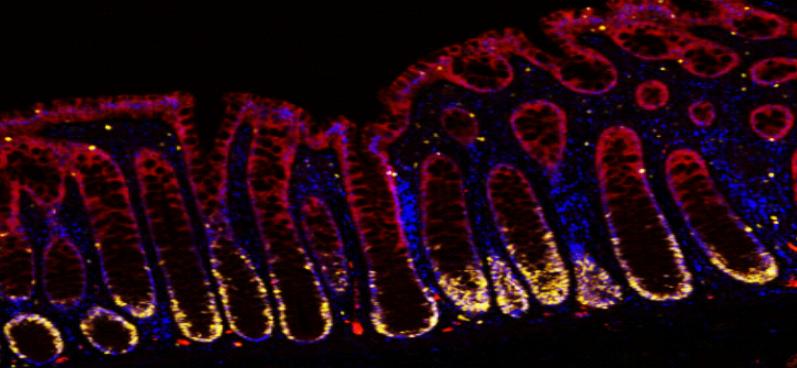

Supplement: Supplementary file 6 — Source data Fig. 4 [file 44319_2025_373_MOESM6_ESM.zip › Figure 4/4J/image_large.PNG]

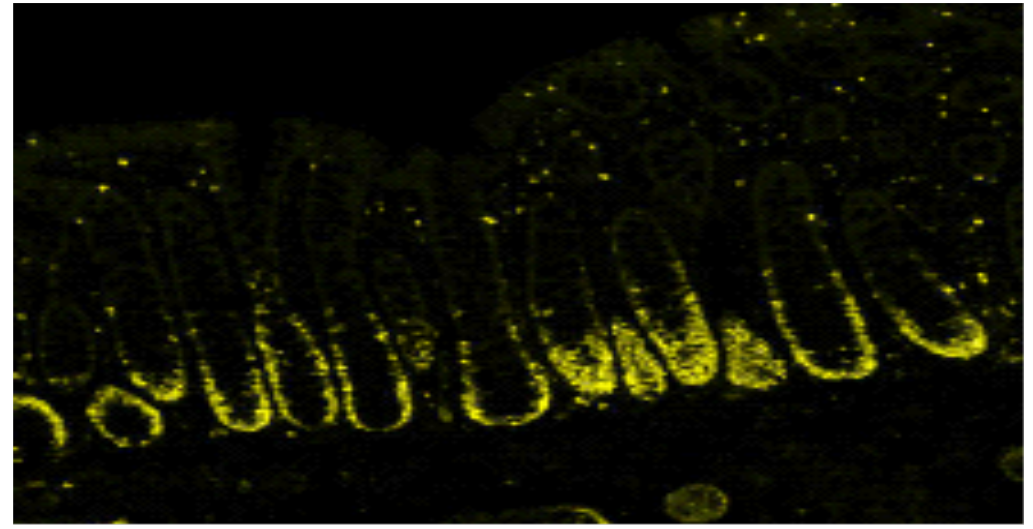

Supplement: Supplementary file 6 — Source data Fig. 4 [file 44319_2025_373_MOESM6_ESM.zip › Figure 4/4J/image_MCM2.PNG]

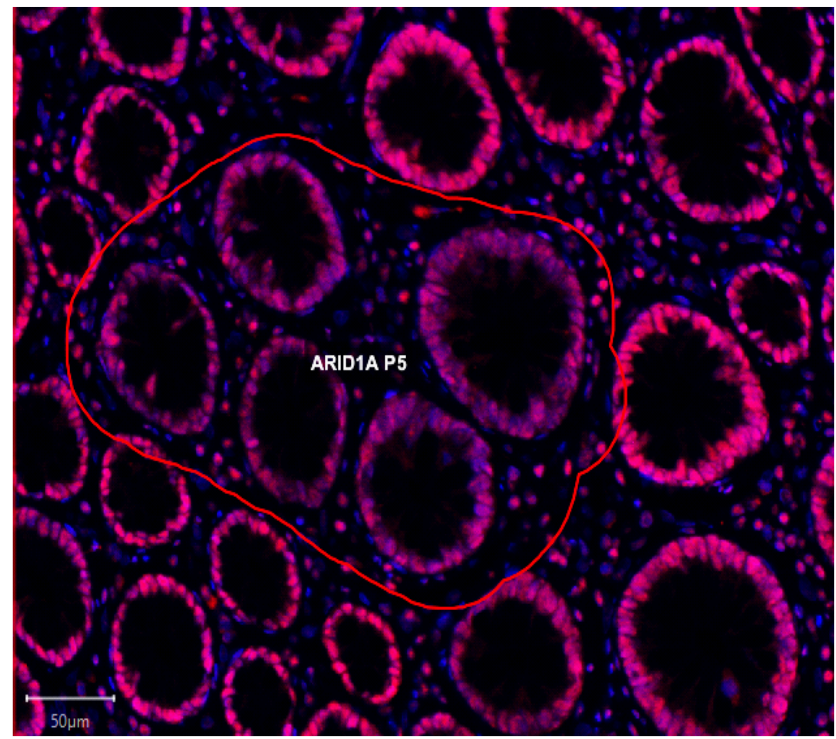

Supplement: Supplementary file 7 — Source data Fig. 5 [file 44319_2025_373_MOESM7_ESM.zip › Figure 5/5A-C/5A_image.PNG]

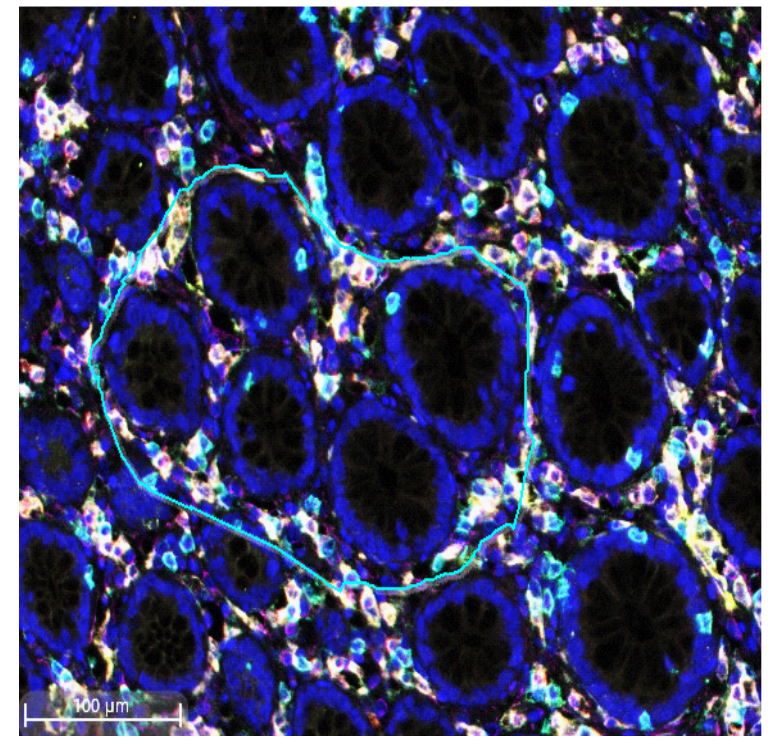

Supplement: Supplementary file 7 — Source data Fig. 5 [file 44319_2025_373_MOESM7_ESM.zip › Figure 5/5A-C/5B_image.PNG]

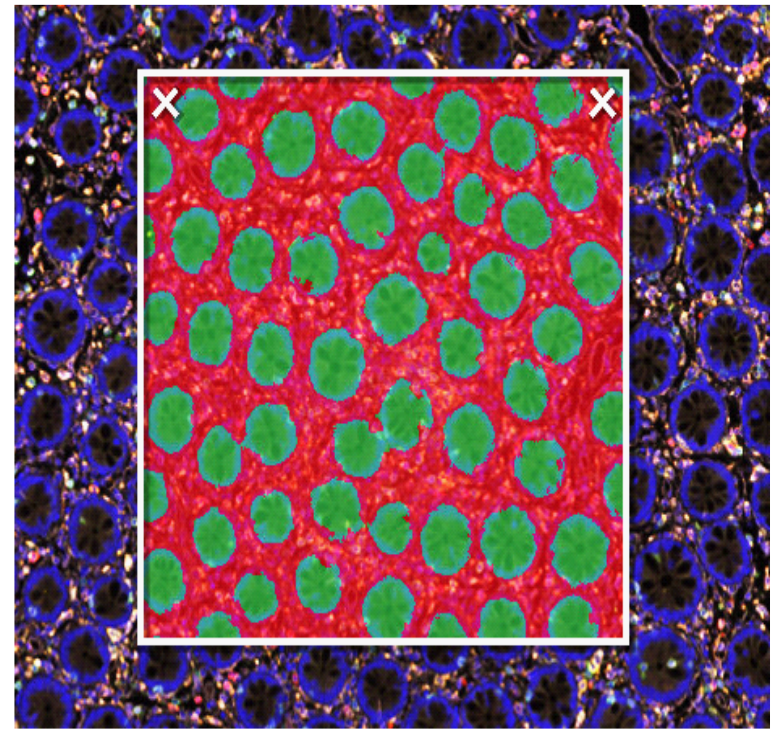

Supplement: Supplementary file 7 — Source data Fig. 5 [file 44319_2025_373_MOESM7_ESM.zip › Figure 5/5A-C/5C_image.PNG]

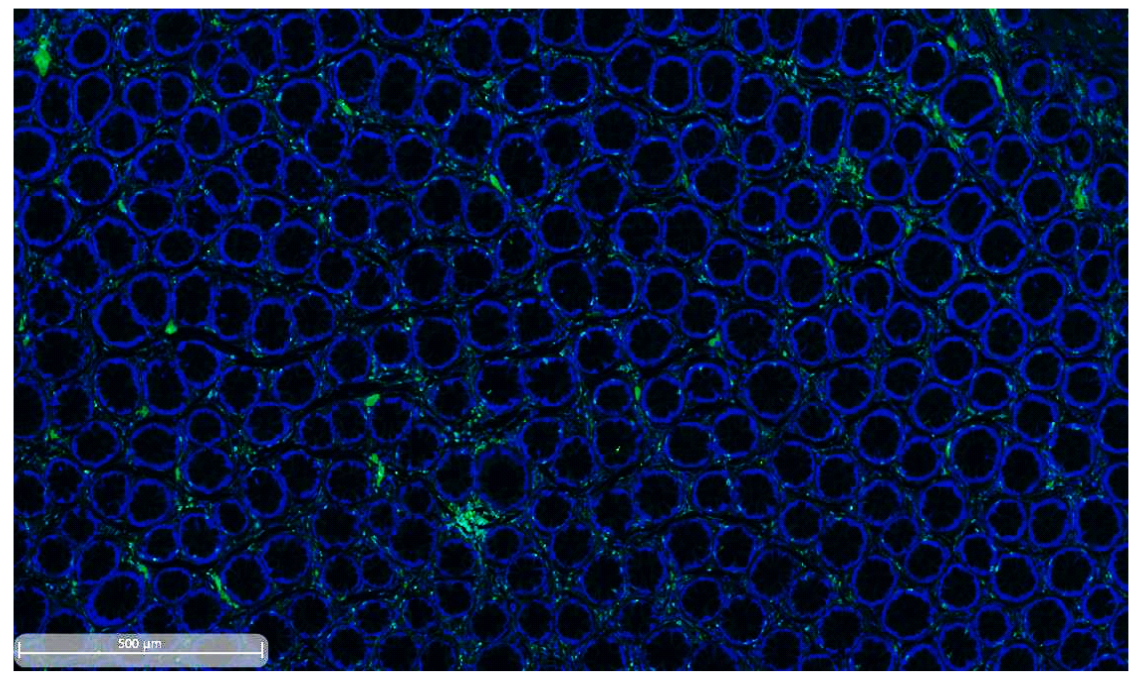

Supplement: Supplementary file 7 — Source data Fig. 5 [file 44319_2025_373_MOESM7_ESM.zip › Figure 5/5N/image_ARID1A-.PNG]

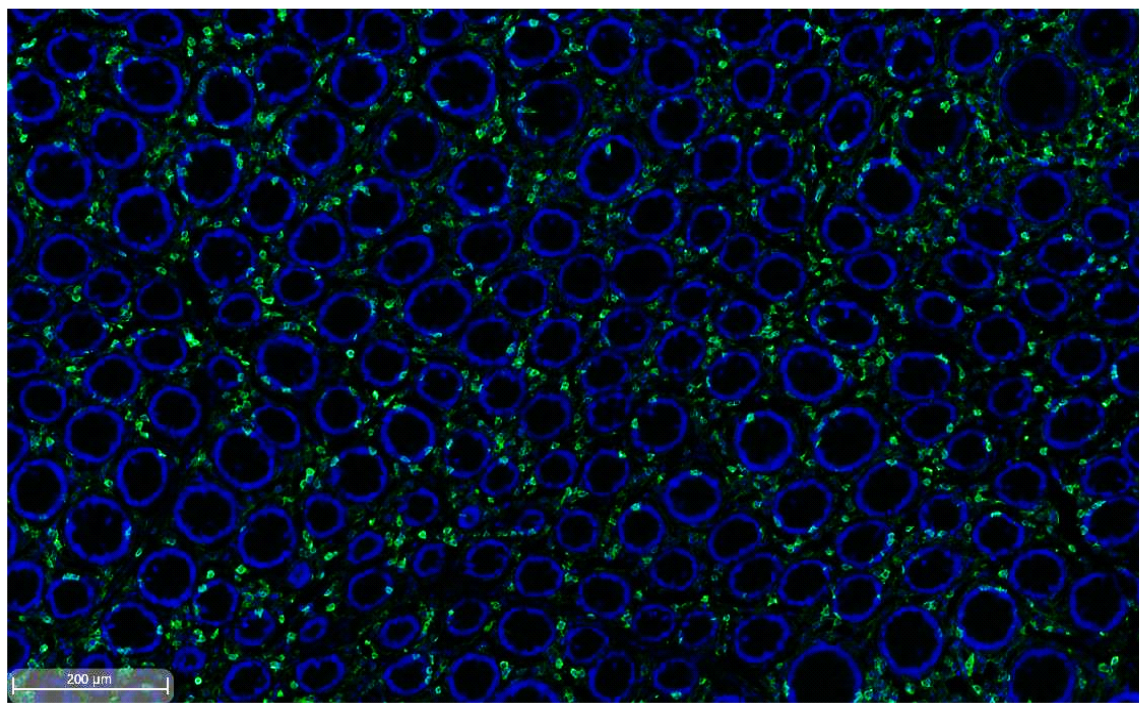

Supplement: Supplementary file 7 — Source data Fig. 5 [file 44319_2025_373_MOESM7_ESM.zip › Figure 5/5N/image_ARID1A+.PNG]
