# Supplementary material for: Haploinsufficient phenotypes promote selection of PTEN and ARID1A-deficient clones in human colon
Source: EMBO Rep. 2025 Feb 7;26(5):1269–89. doi: 10.1038/s44319-025-00373-0 (PMC11893880; doi:10.1038/s44319-025-00373-0)
Supplement: Supplementary file 8 — Expanded View Figures [file 44319_2025_373_MOESM8_ESM.pdf]

Expanded View Figures

Figure EV1. Antibody screen for establishment of tumour suppressor gene as a clonal mark.

(**A**) Failed target- SOX9, not exhibiting uniform expression across the crypt axis. (**B**) Failed target- TCF7L2, identified clone not detected with independent antibody. (**C**) Failed target- FBXW7, antibodies does not separate het and KO samples. VillinCreERT;FBXW7 mouse model used. Human HEK293T cells where FBXW7 was knocked out using CRISPR/Cas9. (**D**) Genetic KO validation of antibodies used for PTEN, SMAD4 and ARID1A. VillinCreERT promoter used for mouse models. (**E**) Independent antibody validation of detected PTEN, SMAD4 and ARID1A clones. Dashed borders within images indicate PTEN, SMAD4 or ARID1A-deficient clones. Data information: All scale bars indicate 50 µm.

© The Author(s)

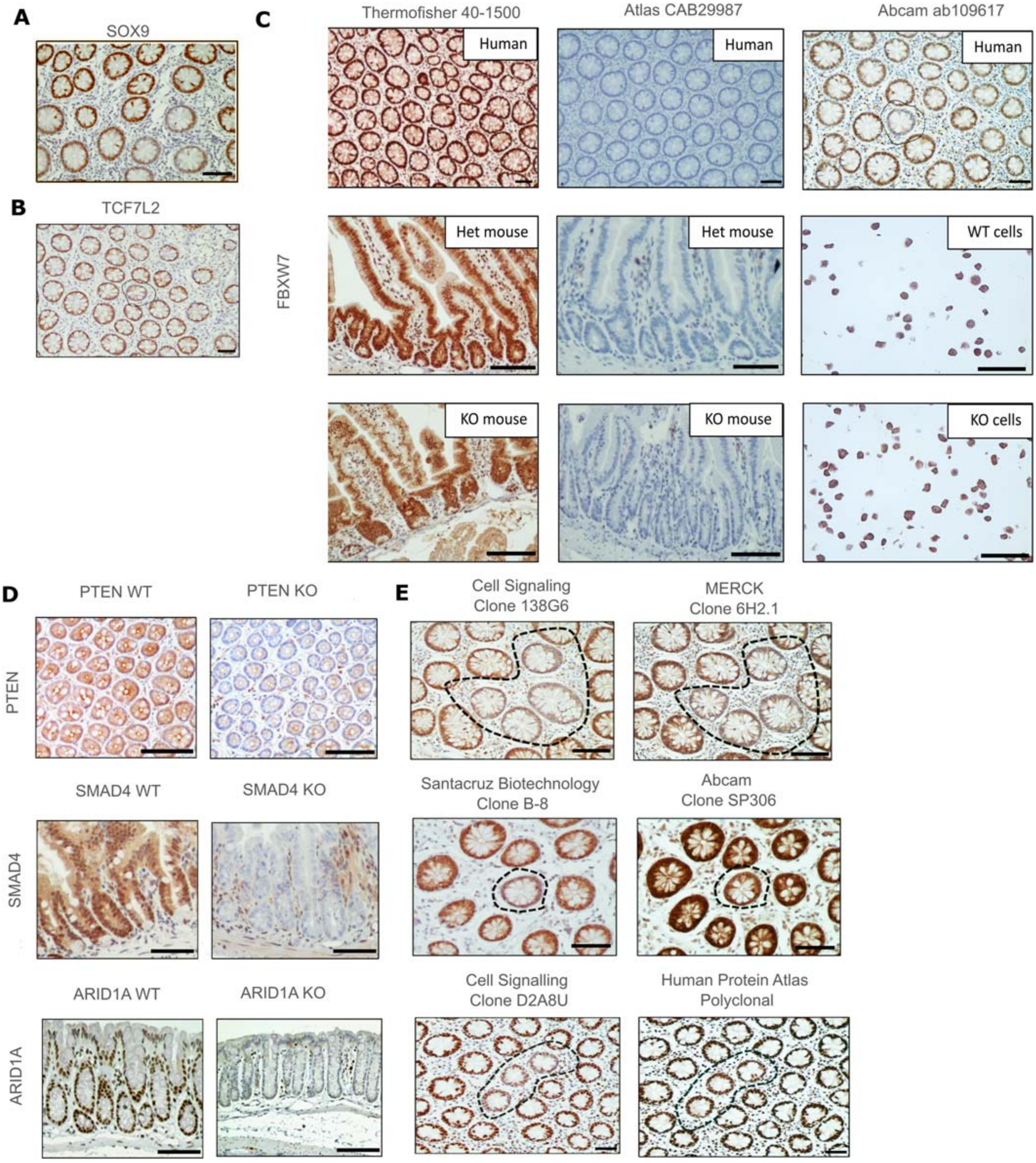
A
SOX9
B
TCF7L2
C
Thermofisher 40-1500
Atlas CAB29987
Abcam ab109617
Human
Human
Human
FBXW7
Het mouse
Het mouse
WT cells
KO mouse
KO mouse
KO cells
D
PTEN WT
PTEN KO
PTEN
SMAD4 WT
SMAD4 KO
SMAD4
ARID1A WT
ARID1A KO
ARID1A
E
Cell Signaling Clone 138G6
MERCK Clone 6H2.1
Santacruz Biotechnology Clone B-8
Abcam Clone SP306
Cell Signalling Clone D2A8U
Human Protein Atlas Polyclonal

 © The Author(s)

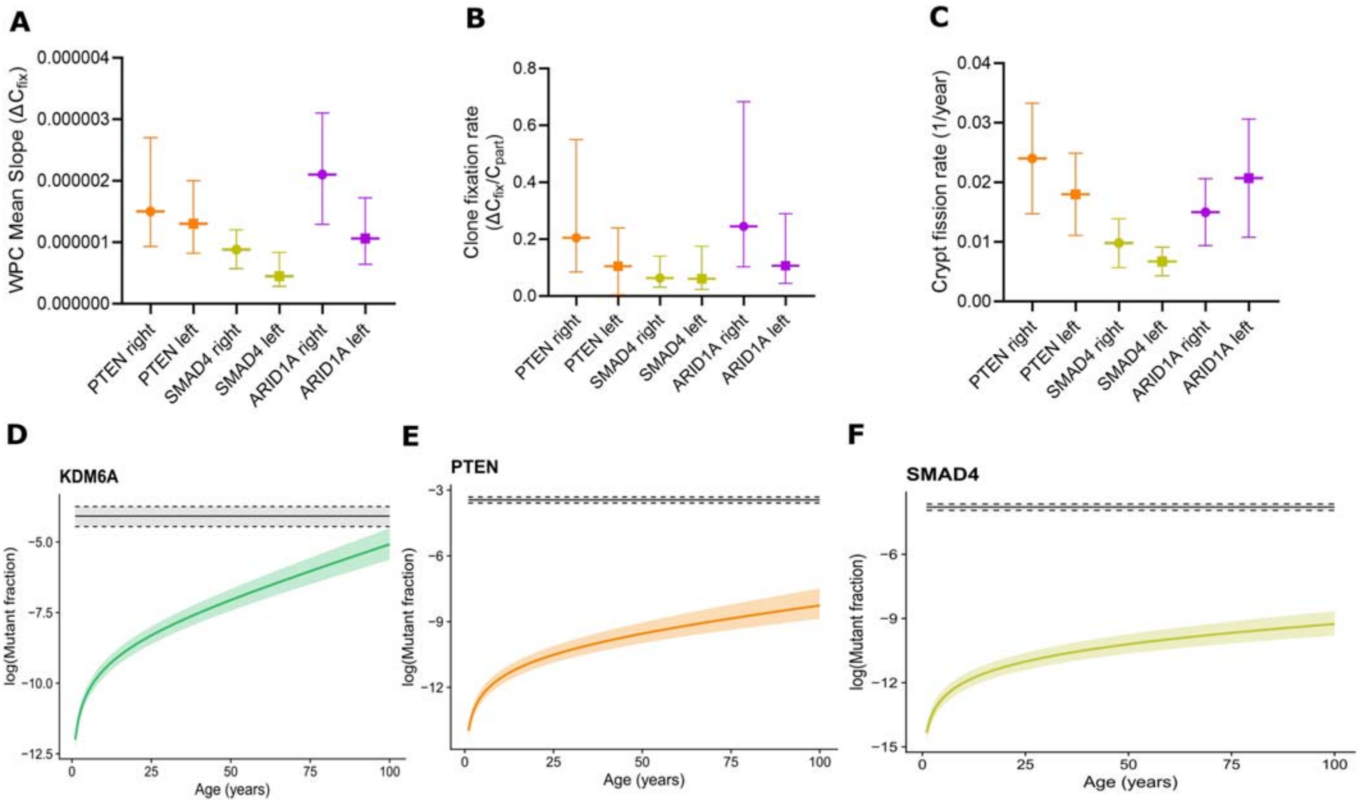

Figure EV2. Clone dynamics and mutational burden.

(**A–C**) Left vs Right side of colon differences. (**A**) Slope (ΔCfix). (**B**) Clone fixation rate (ΔCfix/Cpart). (**C**) Fission rate. (**D**, **E**) Predicted mutational burden in normal tissue based on clone dynamics compared to the frequency of truncating mutations for that gene in colorectal cancer in COSMIC. (**D**) KDM6A, (**E**) PTEN, (**F**) SMAD4. Data information: PTEN: $N = 103$ patients and 2,492,712 total crypts, SMAD4: $N = 88$ patients and 1,661,059 crypts, ARID1A: $N = 101$ patients, $N = 1{,}990{,}179$ crypts. Data presented as mean. Coloured shading indicates 95% credible intervals. Dotted black lines indicate 95% confidence intervals.

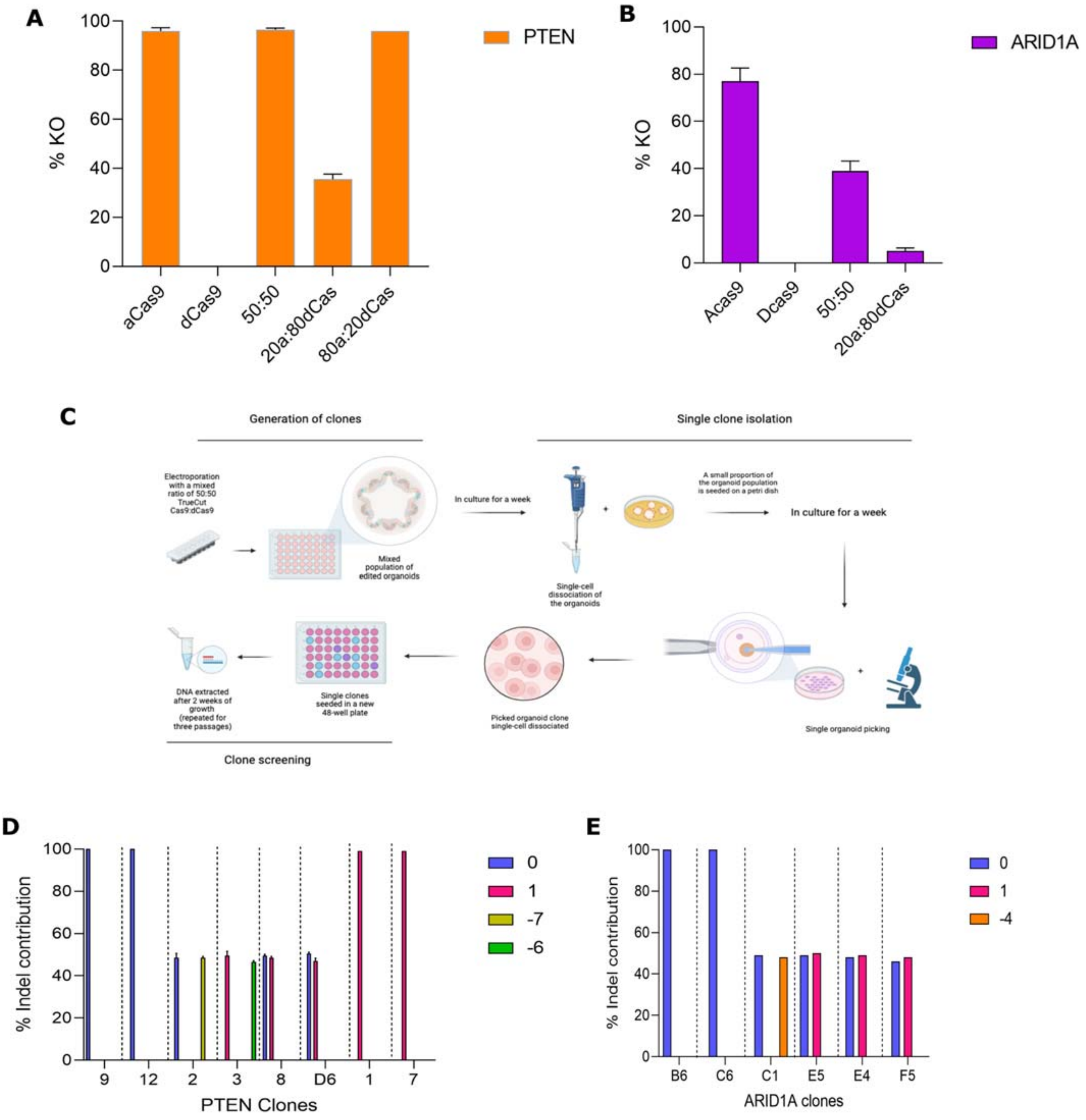

Figure EV3. CRISPR-Cas9 ribonucleoprotein-based editing to generate organoids with heterozygous mutations.

(**A**, **B**) The ratio of active Cas9 (aCas9) to dead Cas9 (dCas9) was trialled for PTEN and ARID1A guides. (**A**) 20a:80dCas ratio was used for PTEN. (**B**) A 50:50 ratio was used for the ARID1A guide. (**A**, **B**) $N = 3$ biological and 2 technical replicates. (**C**) Experimental outline of generation of heterozygous organoids and single clone picking. (**D**) Indel contribution for generated PTEN clones. 0 indicates the WT allele. (**E**) Indel contribution for ARID1A clones. 0 indicates the WT allele. (**D**, **E**) The bar shows individual clones sequenced in forward and reverse directions (error bars). Data information: Data presented as mean ± SD.

 © The Author(s)

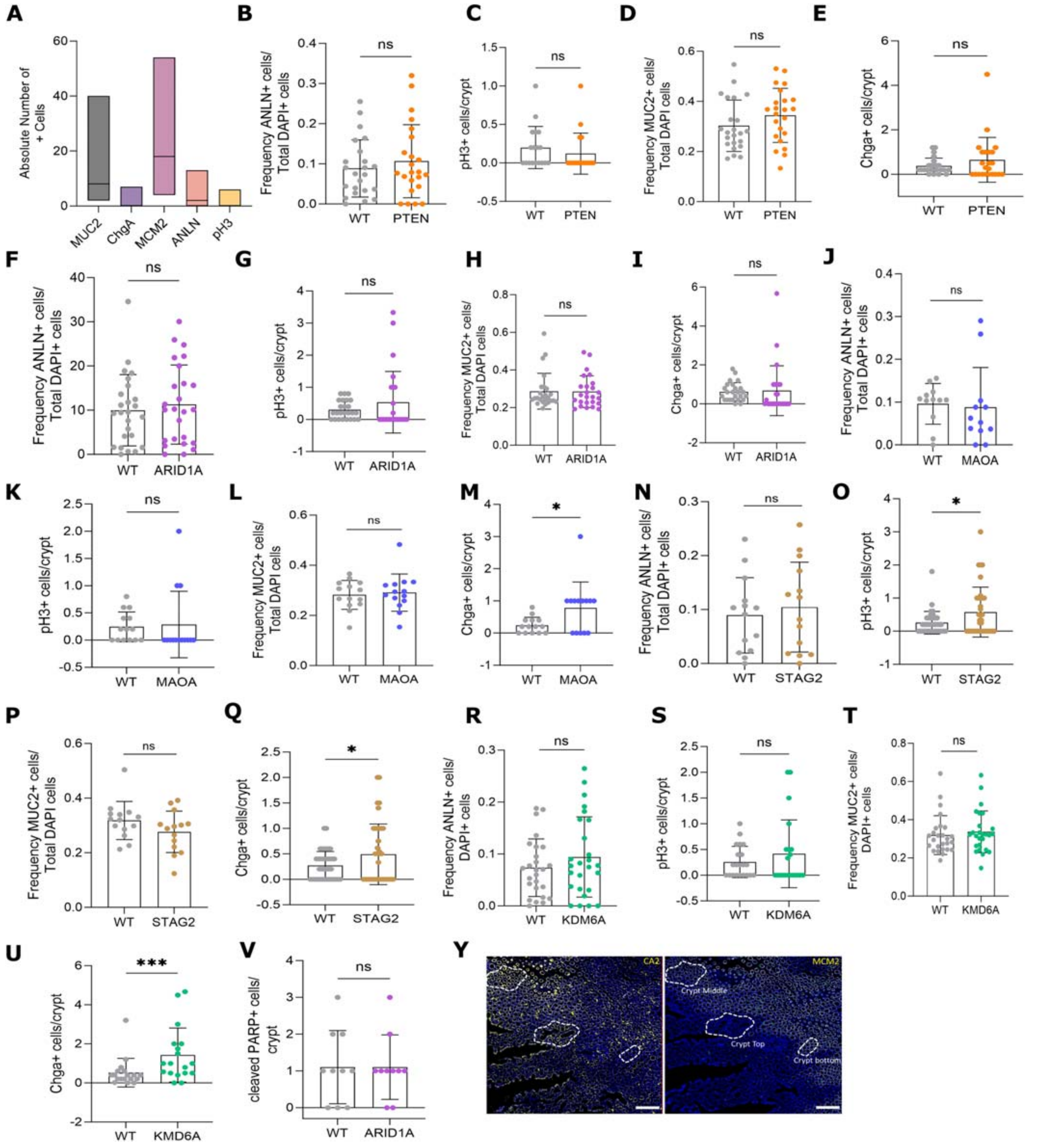
A
Absolute Number of + Cells
MUC2
ChgA
MCM2
ANLN
pH3
B
Frequency ANLN+ cells/ Total DAPI+ cells
ns
WT
PTEN
C
pH3+ cells/crypt
ns
WT
PTEN
D
Frequency MUC2+ cells/ Total DAPI cells
ns
WT
PTEN
E
Chga+ cells/crypt
ns
WT
PTEN
F
Frequency ANLN+ cells/ Total DAPI+ cells
ns
WT
ARID1A
G
pH3+ cells/crypt
ns
WT
ARID1A
H
Frequency MUC2+ cells/ Total DAPI cells
ns
WT
ARID1A
I
Chga+ cells/crypt
ns
WT
ARID1A
J
Frequency ANLN+ cells/ Total DAPI+ cells
ns
WT
MAOA
K
pH3+ cells/crypt
ns
WT
MAOA
L
Frequency MUC2+ cells/ Total DAPI cells
ns
WT
MAOA
M
Chga+ cells/crypt
*
WT
MAOA
N
Frequency ANLN+ cells/ Total DAPI+ cells
ns
WT
STAG2
O
pH3+ cells/crypt
*
WT
STAG2
P
Frequency MUC2+ cells/ Total DAPI cells
ns
WT
STAG2
Q
Chga+ cells/crypt
*
WT
STAG2
R
Frequency ANLN+ cells/ DAPI+ cells
ns
WT
KDM6A
S
pH3+ cells/crypt
ns
WT
KDM6A
T
Frequency MUC2+ cells/ DAPI+ cells
ns
WT
KMD6A
U
Chga+ cells/crypt
***
WT
KMD6A
V
cleaved PARP+ cells/ crypt
ns
WT
ARID1A
Y
CA2
MCM2
Crypt Middle
Crypt Top
Crypt bottom

Figure EV4. Quantification of proliferation and differentiation markers in deficient clones.

(**A**) Number of positive cells per crypt for markers used in the panel. Applied on WT crypts. Box plot shows min, median and max. (**B–U**) ANLN, S/G phase marker. pH3, mitosis marker. MUC2, goblet cells marker. Chga, enteroendocrine marker. Cleaved PARP1, apoptosis marker. ANLN, ph3 and cleaved PARP1 expressed as a number of positive cells per crypt. MUC2 and ANLN expressed as frequency out of total DAPI cells. (**Y**) Definition of crypt axis levels based on CA2 and MCM2 staining in *en face* embedded tissue. Different levels within a section are seen due to tissue not being completely flat during processing. CA2 + MCM2- crypt top. CA2 + MCM2+ crypt middle. CA2− MCM2+ crypt bottom. Dashed borders indicate crypt top, middle or top according to marker expression. Data information: Data presented as mean ± SD. All scale bars indicate 400 µm. A paired *t*-test or Wilcoxon test was performed to assess statistical significance based on the type of distribution. (**B**) $p = 0.297$, (**C**) $p = 0.203$, (**D**) $p = 0.175$, (**E**) $p = 0.355$, (**F**) $p = 0.178$, (**G**) $p = 0.597$, (**H**) $p = 0.56$, (**I**) $p = 0.406$, (**J**) $p = 0.32$, (**K**) $p > 0.999$, (**L**) $p = 0.541$, (**M**) $p = 0.017$, (**N**) $p = 0.36$, (**O**) $p = 0.025$, (**P**) $p = 0.075$, (**Q**) $p = 0.035$, (**R**) $p = 0.12$, (**S**) $p = 0.508$, (**T**) $p = 0.247$, (**U**) $p = 0.0004$, (**V**) $p > 0.999$. *$p < 0.05$, ***$p < 0.001$. (**B–E**) $N = 23$ PTEN and WT clones. (**F–I**) $N = 25$ ARID1A and WT clones. (**J–M**) $N = 12$ MAOA and WT clones. (**N–Q**) $N = 14$ STAG2 and WT clones. (**R–U**) $N = 26$ KDM6A and WT clones. (**V**) $N = 10$ ARID1A and WT clones.

© The Author(s)

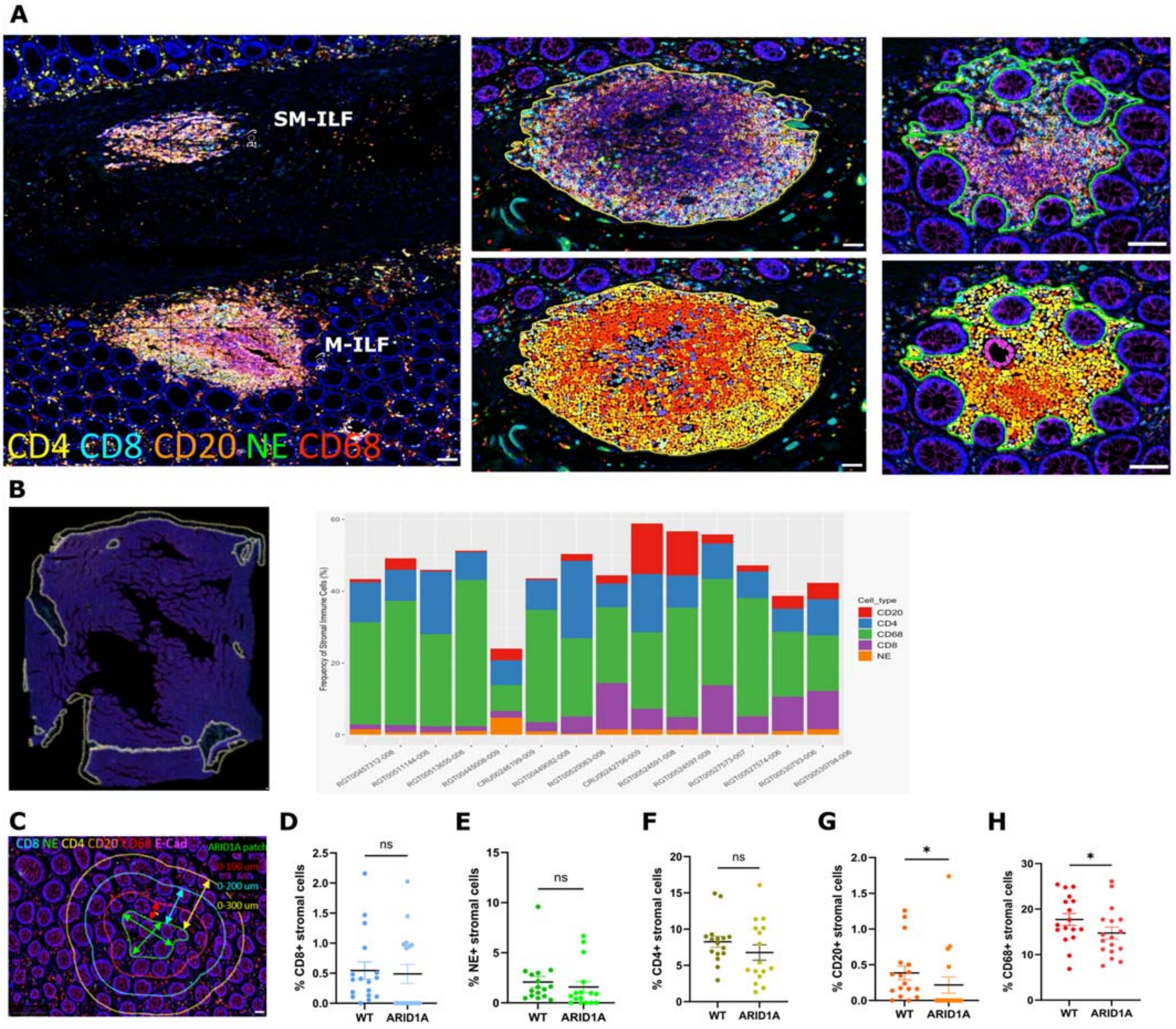

Figure EV5. Characterisation of immune cell populations in the normal human colon.

(**A**) Examples of immune lymphoid follicles in the submucosa (SM-ILF) and the colonic mucosa (M-ILF). The bottom pictures show cell segmentation. (**B**) Whole-section analysis (excluding immune follicles) showing the contribution of different immune cell types. (**C**) Infiltration analysis within and around ARID1A patches. (**D–H**) Percentage of positive stromal cells as defined by trained crypt-stroma random forest classifier (Halo, Indica labs). Cells were measured using the cell segmentation algorithm within the ARID1A patch (>5 crypts) or 300-um radius around the patch (WT). $N = 15$ patients. (**D**) CD8+ cytotoxic T-cells. (**E**) NE+ neutrophils. (**F**) CD4 + T-helper cells. (**G**) CD20 + B-cells. (**H**) CD68+ macrophages. Data information: Data presented as mean ± SD. All scale bars indicate 50 μm except for (**B**) where it indicates 400 μm. A paired Wilcoxon test was performed to assess statistical significance. (**D**) $p = 0.528$, (**E**) $p = 0.175$, (**F**) $p = 0.065$, (**G**) $p = 0.030$, (**H**) $p = 0.027$. *$p < 0.05$.

© The Author(s)
